# Larvae of the genus *Eleodes* (Coleoptera, Tenebrionidae): matrix-based descriptions, cladistic analysis, and key to late instars

**DOI:** 10.3897/zookeys.415.5887

**Published:** 2014-06-12

**Authors:** Aaron D. Smith, Rebecca Dornburg, Quentin D. Wheeler

**Affiliations:** 1 Department of Biological Sciences, Northern Arizona University, PO Box 5640, Flagstaff, AZ, 86011-5640, USA; 2 School of Life Sciences, Arizona State University, PO Box 874501, Tempe, AZ, 85287-4501, USA; 3 SUNY College of Environmental Science and Forestry, 1 Forestry Drive, Syracuse, NY 13210, USA

**Keywords:** Tenebrionidae, larvae, matrix-based descriptions, Eleodes

## Abstract

Darkling beetle larvae (Coleoptera, Tenebrionidae) are collectively referred to as false wireworms. Larvae from several species in the genus *Eleodes* are considered to be agricultural pests, though relatively little work has been done to associate larvae with adults of the same species and only a handful of species have been characterized in their larval state.

Morphological characters from late instar larvae were examined and coded to produce a matrix in the server-based content management system mx. The resulting morphology matrix was used to produce larval species descriptions, reconstruct a phylogeny, and build a key to the species included in the matrix.

Larvae are described for the first time for the following 12 species: *Eleodes anthracinus* Blaisdell, *Eleodes carbonarius* (Say), *Eleodes caudiferus* LeConte, *Eleodes extricatus* (Say), *Eleodes goryi* Solier, *Eleodes hispilabris* (Say), *Eleodes nigropilosus* LeConte, *Eleodes pilosus* Horn, *Eleodes subnitens* LeConte, *Eleodes tenuipes* Casey, *Eleodes tribulus* Thomas, and *Eleodes wheeleri* Aalbu, Smith & Triplehorn. The larval stage of *Eleodes armatus* LeConte is redescribed with additional characters to differentiate it from the newly described congeneric larvae.

## Introduction

Species of the genus *Eleodes* are among the most iconic and recognizable insects of the western United States. Flightless, almost always black in color, and medium to large sized (~10-50 mm), *Eleodes* are perhaps most closely associated with head-standing. While this behavior, linked to the exudation or squirting of a concoction of noxious defensive chemicals from paired reservoirs near the tip of the abdomen, is not unique to *Eleodes*, it has been the source of common names for the genus such as stink or circus beetles.

Larvae of the family Tenebrionidae are known as false wireworms. Feeding on seeds, roots, and subterreanean stems, a number of them are considered agricultural pests, including *Eleodes extricatus* (Say, 1824), *Eleodes hispilabris* (Say, 1824), *Eleodes obsoletus* (Say, 1824), *Eleodes opacus* (Say, 1824), and *Eleodes suturalis* (Say, 1824) (Calkins and Kirk 1975). A summary of species considered agricultural pests and the crops they attack is given by Allsopp (1980). In spite of ecological and agricultural interest in false wireworms, and their potential contribution of a whole suite of characters for phylogenetic and taxonomic studies, knowledge of their morphology, development, and habits remain limited. Doyen (1988) estimated that approximately 240 genera and 300 species of darkling beetle larvae had been described which, as far as we know, remains a reasonable approximation. Of the 190+ currently valid *Eleodes* species, only seven have been previously described in the larval stages.

## Taxonomic history

Relatively few *Eleodes* larvae have been described or characterized (Table 1). Gissler (1878) provided the first larval descriptions in the genus for *Eleodes dentipes* Eschscholtz, 1833 and *Eleodes giganteus* (Mannerheim, 1843). Hyslop (1912) described the larvae of *Eleodes vandykei* Blaisdell, 1909 (then listed as a subspecies of *Eleodes letcheri*) and *Eleodes pimelioides* Mannerheim, 1843 from the Pacific Northwest. McColloch (1918) described *Eleodes tricostatus* (Say), 1824. Wade and St. George (1923) described *Eleodes suturalis* (Say, 1824), followed closely by illustrations, without additional descriptions, of the pygidia of *Eleodes carbonarius* (Say, 1824), *Eleodes opacus* (Say, 1824), and *Eleodes tricostatus* by St. George (1924). Blaisdell (1909) redescribed the larvae of *Eleodes dentipes* in greater detail and described the pupa of *Eleodes clavicornis* Eschscholtz, 1833. The most recent larval description was provided by Thomas (1984) for *Eleodes armatus* LeConte, 1851. In most cases, these descriptions are insufficient to reliably diagnose *Eleodes* larvae to species.

**Table 1.** Previous publications describing or illustrating *Eleodes* larvae.

Species	Publication	Remarks
*Eleodes armatus* (LeConte), 1851	Thomas 1984	egg, larva, and pupa described, larva and pupa imaged
*Eleodes dentipes* (Eschscholtz), 1833	Gissler 1878; Blaisdell 1909	larva briefly described in Gissler (1878); larva redescribed and illustrated in Blaisdell (1909)
*Eleodes giganteus* (Mannerheim), 1843	Gissler 1878	egg and larva characterized; larva illustrated
*Eleodes opacus* (Say), 1824	St. George 1924	pygidium imaged; no description
*Eleodes pimelioides* (Mannerheim), 1843	Hyslop 1912	egg, larva, and pupa described; pygidium of larva imaged
*Eleodes suturalis* (Say), 1824	Wade and St. George 1923	egg, larva, and pupa described, larval natural history discussed, egg and pupa imaged
*Eleodes tricostatus* (Say), 1824	McColloch 1918; St. George 1924	egg, larva, and pupa briefly characterized, larval natural history discussed; right mandible and pygidium of larva imaged in St. George (1924)
*Eleodes vandykei* Blaisdell, 1909	Hyslop 1912	egg, larva, and pupa described, egg, larva, and pupa imaged; species listed as *Eleodes letcheri vandykei*

## Matrix-based taxonomy

A number of modern taxonomic works on insects have produced descriptions based on matrices of morphological characters, including Winterton (2009), Yoder et al. (2009), Talamas et al. (2011), and Mullins et al. (2012) to name a few. The advantages of this methodology include a structured and explicit differentia between the character states exhibited by each species or other taxonomic units, the ability to score new species into the matrix, an option to further utilize the matrix for phylogenetic analyses, and the capability to turn a matrix into a multi-entry key or link it to other data sources, such as an anatomy ontology. The present work is intended as a first step to describe the larvae of the genus *Eleodes*, define important characters for species and subgeneric differentiation, provide a first glimpse into evolutionary relationships within the genus, and provide a platform to link character states to the developing Coleoptera Anatomy Ontology (ColAO).

## Methods and Materials

**Morphological parameters.** Measurements were taken using either digital calipers, an optical micrometer attached to a Nikon SMZ 1500 stereomicroscope, or measurement scales set in Photoshop specific to the camera and lens used to take measurements from images. Total length (TL) was measured from the anterior edge of the clypeus to the dorsomedial apex of abdominal segment IX. Prothoracic width (PW) and length (PL) were measured dorsally across the widest and longest points on the segment respectively, head capsule width (HW) was measured dorsally across the widest portion of the head (generally near the apex of the cranial stem). Terminology primarily follows Lawrence (1991). Dissections were performed using fine forceps and a sharpened #0 insect pin.

Photographs of specimens or characters were taken using a BK Plus or Passport Imaging system (R. Larimer, www.visionarydigital.com). Montaged images were assembled using Zerene Stacker (zerenesystems.com/stacker/) and backgrounds were cleaned up in Adobe Photoshop CS5. Confocal laser images were taken on a Zeiss LSM 710.

**Rearing.** Adult *Eleodes* specimens were hand collected from throughout the southwestern United States. Specimens were maintained in separate plastic containers for each species, locality, and collecting event on a substrate of sand. Every one to two weeks, containers were sifted for eggs and larvae. Larvae were reared on a sand/food substrate in plastic containers, with either plaster of Paris at the bottom watered though a vinyl tube to maintain a moisture gradient (Brown 1973) or with daily watering. A study detailing rearing regimes, instar numbers, and life histories for the reared species is forthcoming (Dornburg, Smith & Wheeler, in preparation).

**Matrix-based descriptions.** To allow for easier direct comparisons between larvae of different species and provide a framework for the addition of larvae from more *Eleodes* species in the future, descriptions were produced from a morphological character matrix and edited for traditional telegraphic description format. The character matrix was built in mx (Yoder et al. 2010), based on 86 morphological characters (Appendices 1 and 2). Mx was also used to produce the initial descriptions. Single state characters included in the descriptions, were also included in the matrix. All specimens scored in the matrix were classified as late (7th–11th) instar larvae based on their size or observed number of molts. The one exception was *Eleodes caudiferus*, in which only third instar larvae were available.

**Phylogeny.** A modified subset of the morphology matrix consisting of 48 characters scored for 13 species of *Eleodes* larvae, plus two outgroup species (*Tenebrio molitor* Linnaeus and *Zophobas morio* (Fabricius)), was exported to TNT (Goloboff et al. 2008) and Winclada-NONA (Nixon 2002, Goloboff 1999) for phylogenetic analyses. Invariant characters and characters judged to be potentially highly variable between specimens (e.g. many characters involving color) were excluded from the analyses. Some character states were reordered and/or combined in the matrix used for analyses (Appendices 3 and 4) to reflect outgroup scoring and to clarify discrete parsimony-informative states. Characters and states from Appendices 3 and 4 are abbreviated in the text as (character:state).

Traditional searches were run with 10,000 random additions and TBR branch swapping. New technology searches were also performed using a variety of settings for the Sectorial Search, Rachet, Drift, and Tree fusing functions. Standard bootstrap (10,000 replicates) and Bremer support were assessed in TNT.

## Results

**Figure 1.**
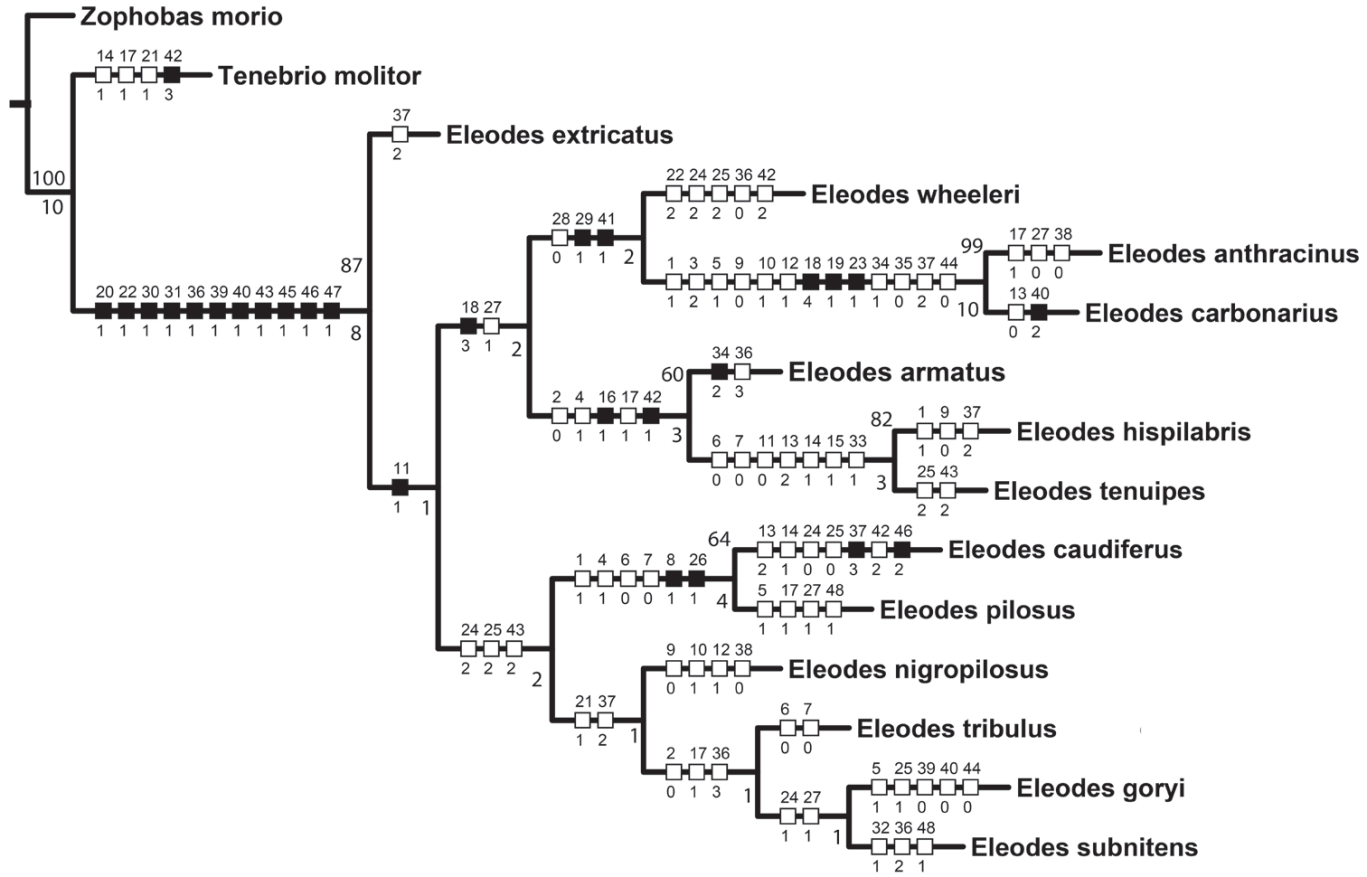
Most parsimonious tree (L = 141, CI = 0.5, RI = .53) based on larval morphology. Numbers not associated with rectangles are bootstrap support values (above branches) and Bremer support values (below branches). Smaller numbers above rectangles on branches represent character number; numbers below rectangles represent character state. Black rectangles correspond to non-homoplasious character state changes. White rectangles correspond to homoplasious character state changes. All character states were unambiguously optimized on the tree.

The phylogenetic analyses returned one most parsimonious tree (Fig. 1). The genus *Eleodes* was relatively strongly supported (BS = 87, Bremer = 8). *Eleodes extricatus* was placed at the base of the genus with the rest of the *Eleodes*, excluding a reversal in *Eleodes hispilabris* + *Eleodes tenuipes*, having moderately punctate clypei (11:1). While the backbone of the clade had little support, several groupings were supported in the analyses.

*Eleodes carbonarius* + *Eleodes anthracinus*, representing the only members of the subgenus *Melaneleodes* in the analyses, was well supported (BS = 99, Bremer = 10). The presence of four long setae on the ligula (18:4, Fig. 11A) and a trapezoidal hypopharyngeal sclerome (19:1; Fig. 12A) may represent synapomorphies for the subgenus.

*Eleodes armatus* + (*Eleodes tenuipes* + *Eleodes hispilabris*) was supported (BS = 60, Bremer = 3), and represents most of the members of the nominate subgenus *Eleodes* in the analyses. The three species share two synapomorphies within the species sampled. One, the arrangement of anterior sensory papillae (16:1, Fig. 9B–C); and two, the presence of a distinct apical tooth on the pygidium (42:1, Fig. 14A). *Eleodes caudiferus*, another species currently in the nominate subgenus, is lacking both characters and was (BS = 64, Bremer = 4) supported in a sister relationship with *Eleodes pilosus* from the subgenus *Tricheleodes*. Both *Eleodes caudiferus* and *Eleodes pilosus* adults are found on sand dunes, and the two larval synapomorphies the species share in the matrix (8:1 and 26:1) are based on the presence of dense setation, a common adaptation to living on sand. Hence, it is possible these character codings represent convergence based on larval habitat. *Eleodes caudiferus* also had one unusual autapomorphy in the presence of longitudinal tomentose bands of setae along the margins of the abdominal sternites (Fig. 13A), which may also be an adaptation for living primarily on unconsolidated dunes. *Eleodes tribulus* was suggested as a member of the nominate subgenus (Triplehorn and Aalbu 1987), but also lacks the two synapomorphies found in other species of the subgenus. It was weakly supported in a relationship with *Eleodes goryi* + *Eleodes subnitens* from the subgenus *Promus*. Determining whether *Eleodes caudiferus* and *Eleodes tribulus* belong in the subgenus *Eleodes* requires further analyses of additional data.

### Larval descriptions

Larvae are described or redescribed to include differential characters to separate species within the genus. Verbatim locality label data are listed with “/” indicating line breaks on the label.

#### Eleodes

Eschscholtz, 1829

http://species-id.net/wiki/Eleodes

##### Material examined.

Over 1,400 larval *Eleodes* specimens were examined for this study from 14 *Eleodes* species. In addition, historical descriptions and *Eleodes* specimens for which the species could not be confirmed due to a lack of positive association between adults and larvae also conform to the generic description provided.

##### Description.

**Figure 2.**
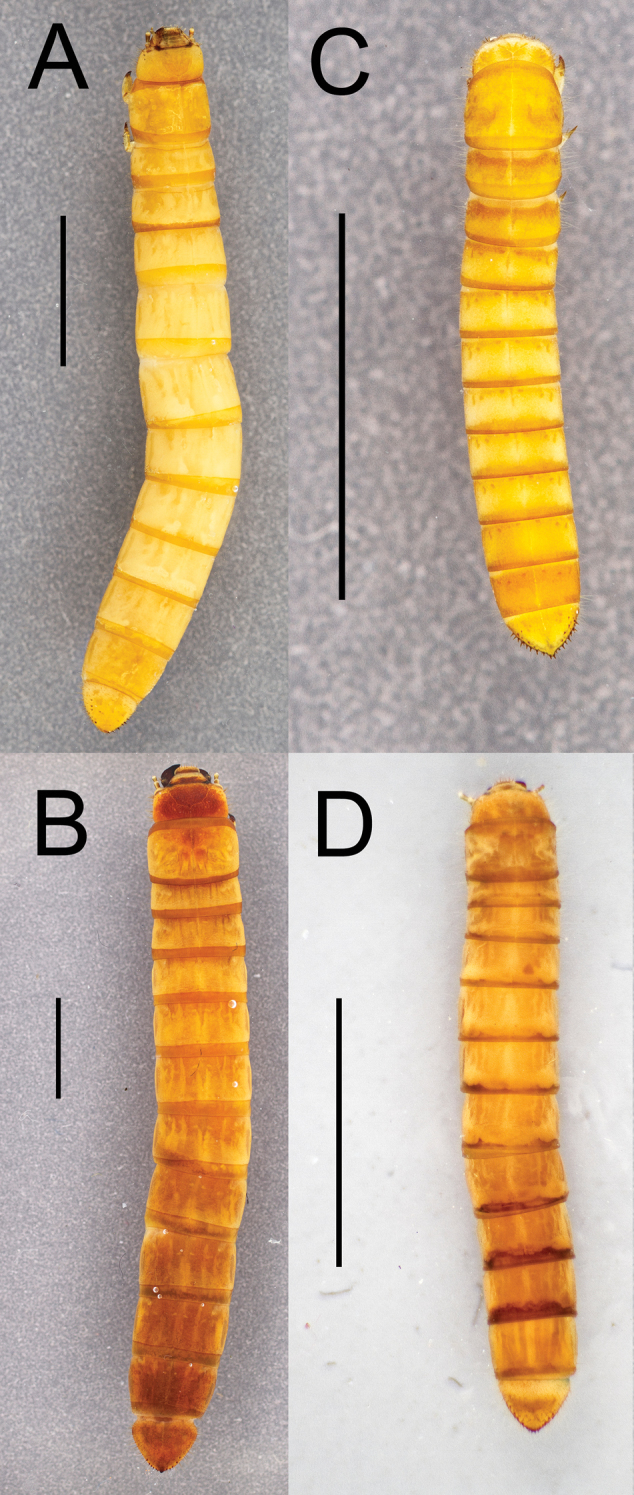
Dorsal habitus of four *Eleodes* species: **A** *Eleodes (Caverneleodes) wheeleri*; **B** *Eleodes (Eleodes) armatus* **C** *Eleodes (Eleodes) caudiferus* **D** *Eleodes (Eleodes) tribulus*. Scale bar = 5 mm.

**Figure 3.**
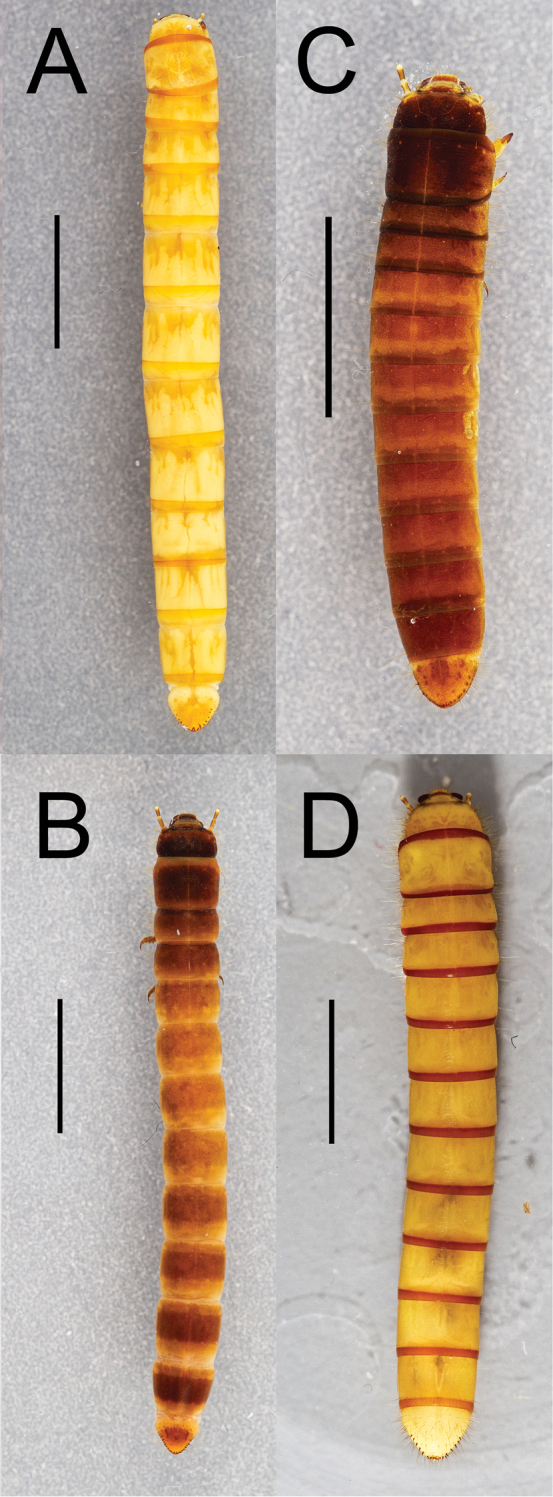
Dorsal habitus of four *Eleodes* species: **A** *Eleodes (Litheleodes) extricatus* **B** *Eleodes (Melaneleodes) anthracinus* **C** *Eleodes (Melaneleodes) carbonarius* **D** *Eleodes (Tricheleodes) pilosus*. Scale bar = 5 mm.

**Figure 4.**
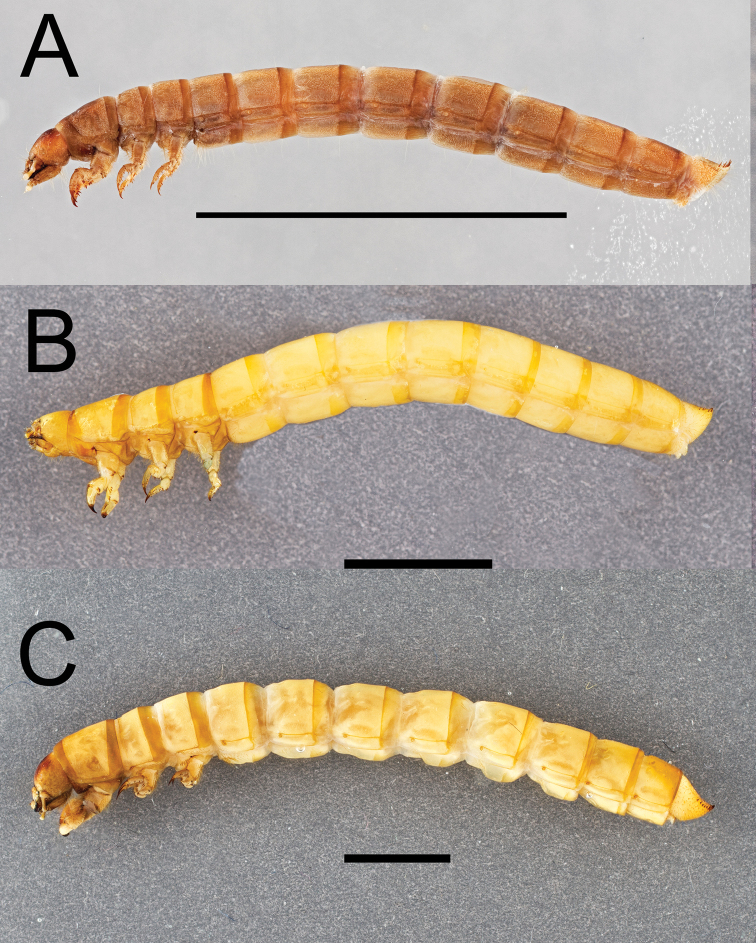
Lateral habitus of three *Eleodes* species: **A** *Eleodes (Blapylis) nigropilosus* **B** *Eleodes (Caverneleodes) wheeleri* **C** *Eleodes (Eleodes) armatus*. Scale bar = 5 mm.

**Figure 5.**
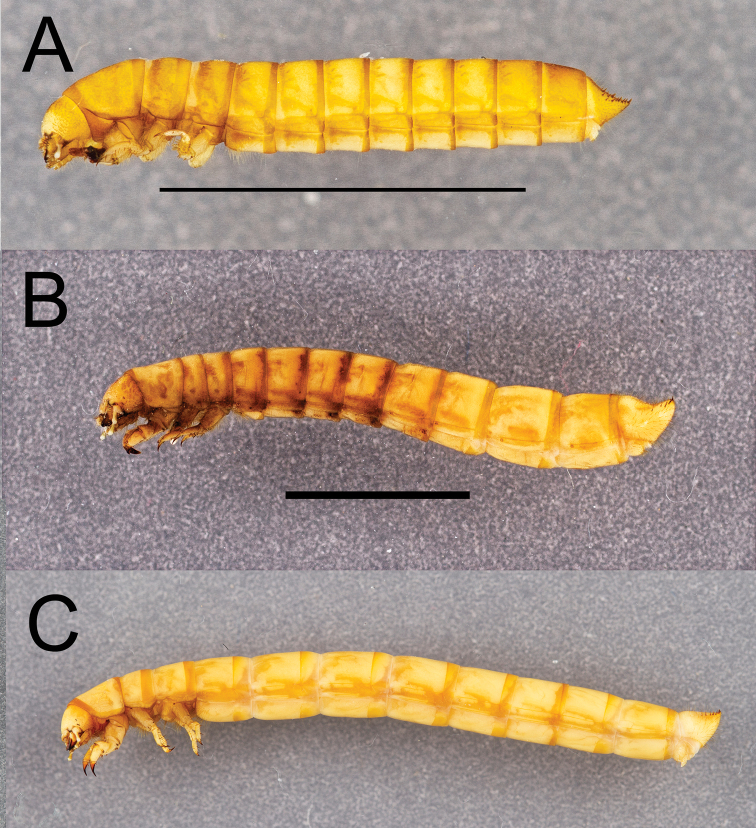
Lateral habitus of three *Eleodes* species: **A** *Eleodes (Eleodes) caudiferus* **B** *Eleodes (Eleodes) tribulus* **C** *Eleodes (Litheleodes) extricatus*. Scale bar = 5 mm.

**Figure 6.**
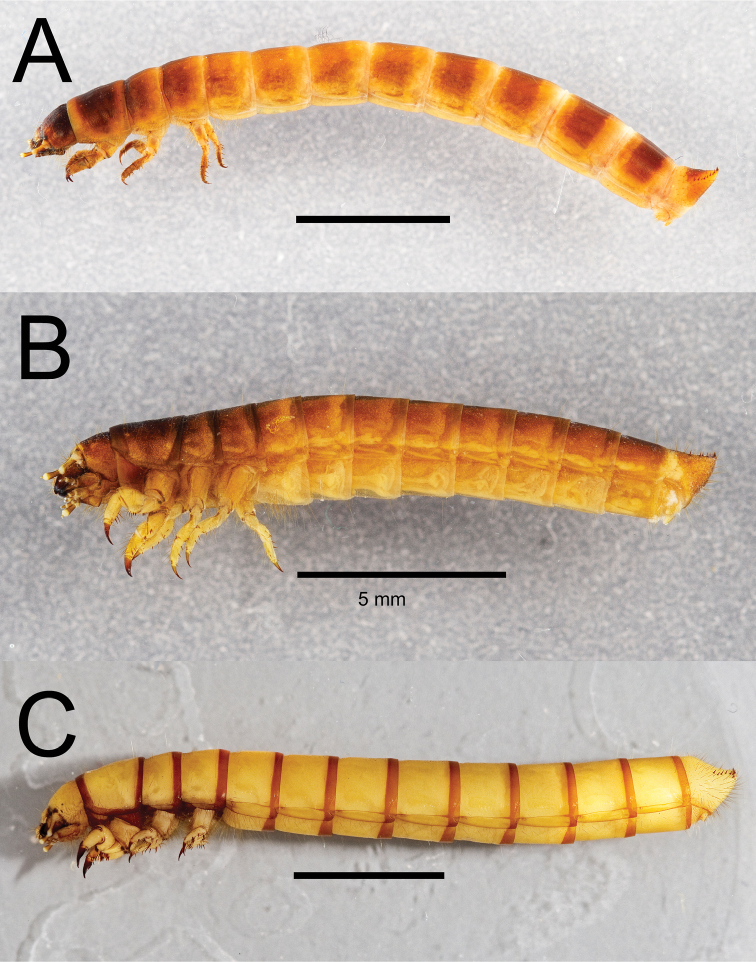
Lateral habitus of three *Eleodes* species: **A** *Eleodes (Melaneleodes) anthracinus* **B** *Eleodes (Melaneleodes) carbonarius* **C** *Eleodes (Tricheleodes) pilosus*. Scale bar = 5 mm.

Integument strongly sclerotized, light tan to nearly black in color; setose, with hair-like setae throughout and spinose setae on legs and abdominal tergite IX. Thoracic and abdominal segments subcylindrical, surface coriaceous (Figs 2A–D, 3A–D, 4A–C, 5A–C, 6A–C).

**Figure 7.**
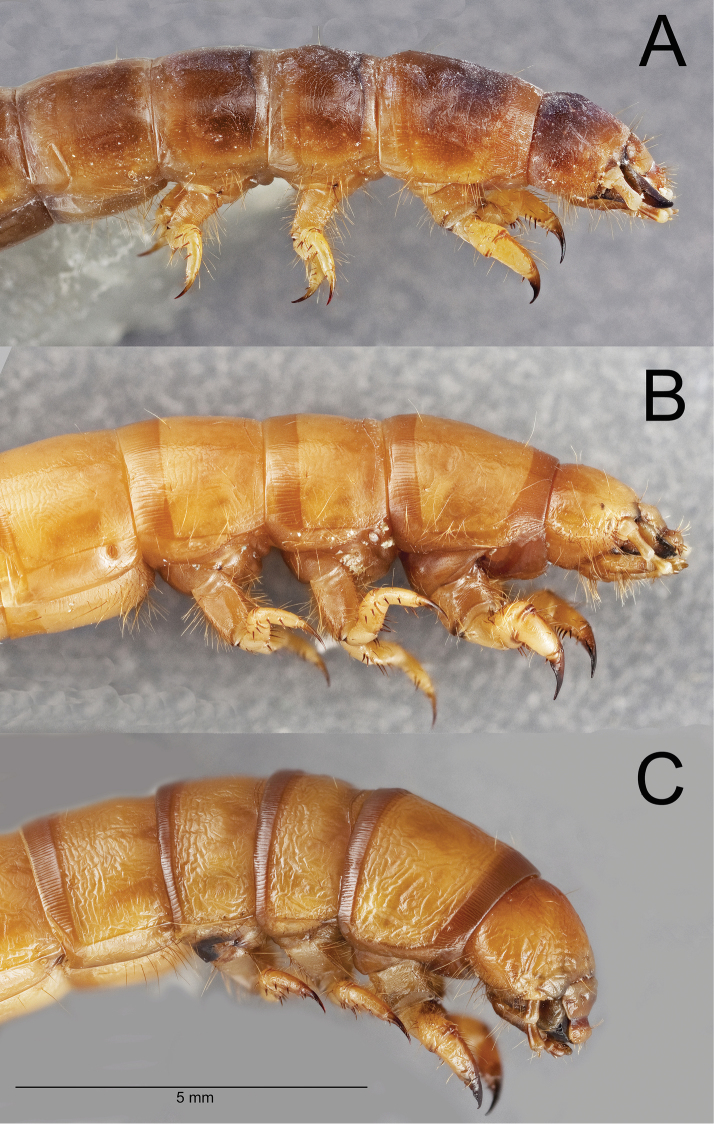
Lateral habitus of the head and thoracic segments of three *Eleodes* species: **A** *Eleodes (Melaneleodes) anthracinus* **B** *Eleodes (Litheleodes) extricatus* **C** *Eleodes (Promus) subnitens*. Scale bar = 5 mm.

**Figure 8.**
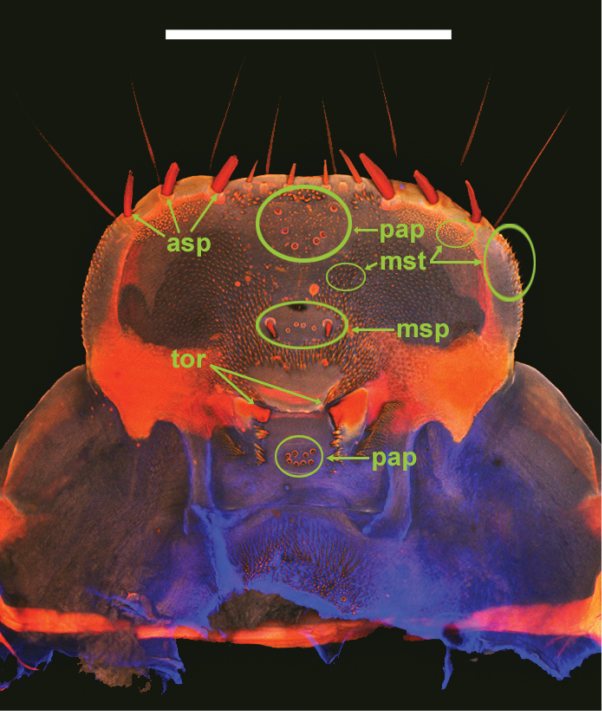
*Eleodes (Melaneleodes) anthracinus*, epipharnyx. asp = anterior spines, msp = medial spines, mst = microsetae, pap = sensory papillae, tor = tormae. Scale bar = 1 mm.

**Figure 9.**
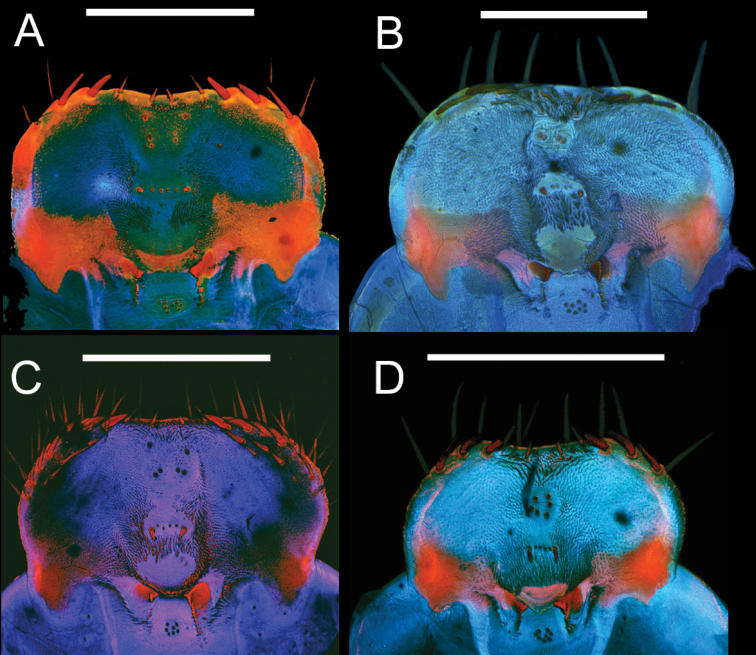
Epipharynges of four *Eleodes* species: **A** *Eleodes (Melaneleodes) carbonarius* **B** *Eleodes (Eleodes) armatus* **C** *Eleodes (Eleodes) hispilabris* **D** *Eleodes (Eleodes) tribulus*. Scale bars = 1 mm.

**Figure 10.**
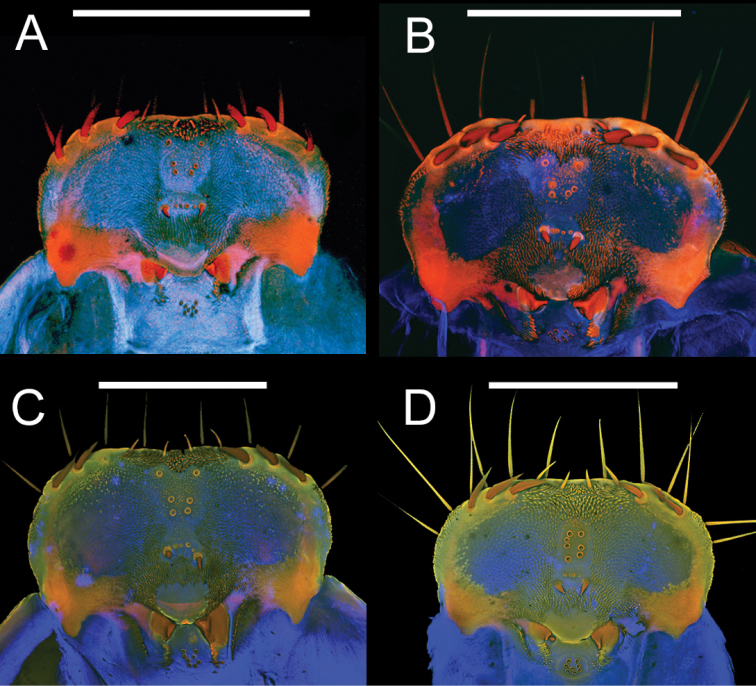
Epipharynges of four *Eleodes* species: **A** *Eleodes (Litheleodes) extricatus* **B** *Eleodes (Promus) goryi* **C** *Eleodes (Promus) subnitens* **D** *Eleodes (Tricheleodes) pilosus*. Scale bars = 1 mm.

**Figure 11.**
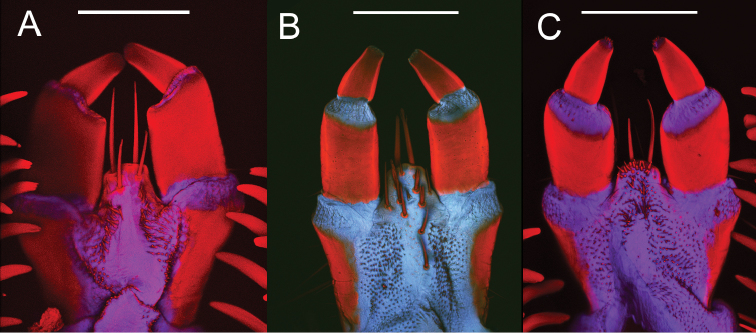
Ligulas of three *Eleodes* species: **A** *Eleodes (Melaneleodes) carbonarius* **B** *Eleodes (Eleodes) armatus* **C** *Eleodes (Promus) goryi*. Scale bars = 200 µm.

**Figure 12.**
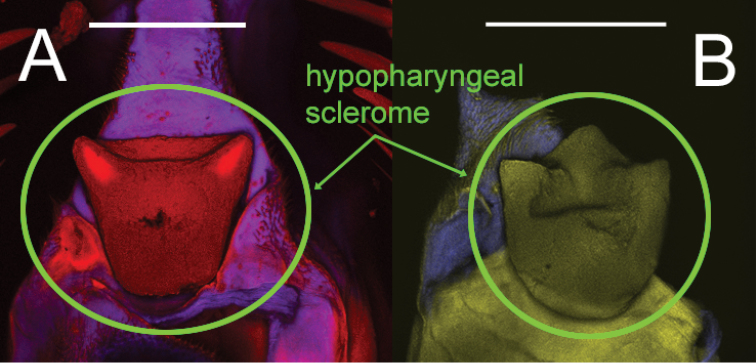
Hypopharyngeal scleromes of two *Eleodes* species: **A** *Eleodes (Melaneleodes) carbonarius* **B** *Eleodes (Litheleodes) extricatus*. Scale bars = 200 µm.

**Head.** Prognathous or slightly declined (Fig. 7A–C), weakly dorsoventrally flattened, strongly constricted before occipital foramen. Epicranial stem one-fourth to one third head capsule length; frontal arms U-shaped or sinuate, occasionally obscured by sculpturing. Frons and dorsal portion of epicranial plates weakly to moderately rugose; punctate, punctures minute, lacking setae. Ventrolateral portions of epicranial plates setose; setae golden, erect; two stemmata present on each plate, pigmented spots often faded. Clypeus trapezoidal, often weakly transversely raised medially. Labrum with two transverse rows of six to fourteen erect setae present medially and subapically; anterior margin straight or weakly emarginate. Epipharynx (Figs 8, 9A–D, 10A–D) with stout spiniform setae along anterior margin, an anterior cluster of four to six variably arranged spinules, a subanterior transverse row of four small spinules subtended by two spinose setae and posterior cluster of six to eight small spinules; tormae symmetrical or asymmetrical. Mandible apex bidentate, mola concave. Ligula small, setation variable (Fig. 11A–C). Hypopharyngeal sclerome pentagonal or trapezoidal (Fig. 12A–B). Gula distinct, trapezoidal, widest in basal half. Antenna three segmented, cylindrical.

**Thorax.** Prothoracic tergum 1.2× or more length of meso- or metaterga (Figs 2A–D, 3A–D); anterior transverse striated band present, generally darker than protergal disc; lateral margins with granulated band either distinct or barely visible (Fig. 7A–C). Posterior transverse striated band present on all thoracic tergites. Meso- and metathoracic tergites wider than long. Mesothoracic spiracle simple, ovate, approximately 1.5× size of abdominal spiracles; reduced metathoracic spiracle visible, less than one-fourth size of mesothoracic spiracle. Legs. Prothoracic leg slightly longer, much thicker than meso- and metathoracic legs; prothoracic tarsungulus strongly sclerotized, sickle-shaped; dorsal surface of prothoracic femur with faintly indicated basal sclerotized band; dorsal surface of prothoracic tibia slightly more sclerotized than ventral surface.

**Figure 13.**
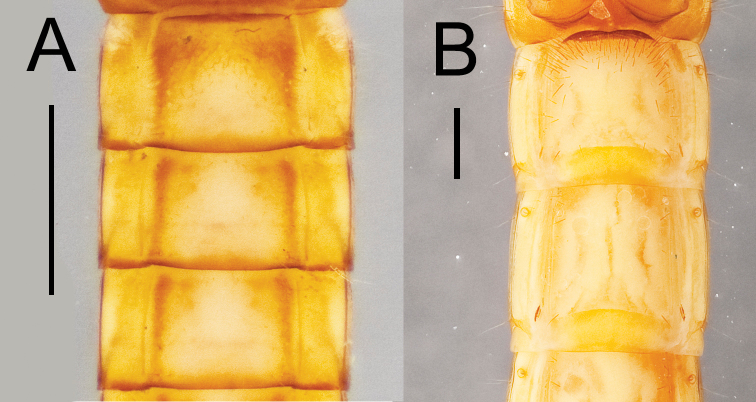
Abdominal sternites I and II for two *Eleodes* species: **A** *Eleodes (Eleodes) caudiferus* **B** *Eleodes (Litheleodes) extricatus*. Scale bars = 1 mm.

**Figure 14.**
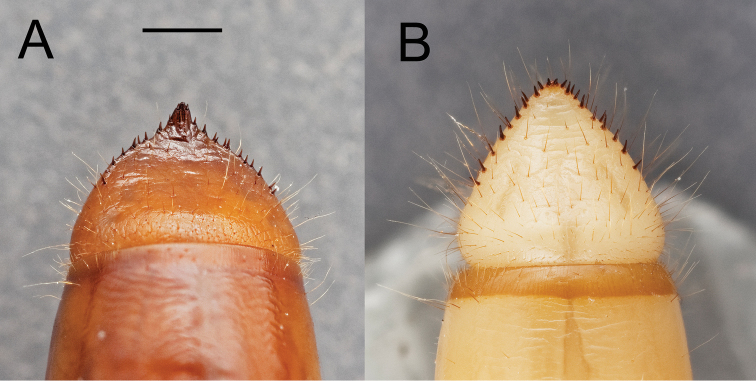
Pygidia of two *Eleodes* species: **A** *Eleodes (Eleodes) hispilabris* **B** *Eleodes (Tricheleodes) pilosus*. Scale bar = 1 mm.

**Abdomen.** Abdominal tergites and sternites I–VIII with transverse striated bands present along posterior margins. Abdominal sternite I setose (Fig. 13A–B). Abdominal segment IX (pygidium) triangular in dorsal view, gradually reflexed to apex, urogomphi absent, apical tooth present or absent (Fig. 14A–B); marginal row of socketed spines present around posterior two-thirds to one half of segment. Abdominal segment X located ventrally; pygopods short, subconical, each with erect setae.

##### Variation.

*Eleodes* larvae can vary greatly in pigmentation, size, number of spines on the legs and pygidium, and the overall degree of sclerotization. Characters in the matrix relating to general integument coloration (6, 24, 45, 46, 47, 69) can vary greatly between specimens depending on age of specimen, length of time since last instar, and preservation method. There may also be genetic variation, though specimens from our populations were generally homogeneous.

##### Diagnosis.

All known *Eleodes* larvae share the following combination of characters: head capsule weakly dorsoventrally flattened, strongly constricted before occipital foramen; prothoracic tergum 1.2× or more length of meso- or metaterga, anterior transverse striated band present, lateral margins with granulated band either distinct or barely visible; prothoracic leg slightly longer and much thicker than meso- and metathoracic legs; 8–38 socketed spines on the pygidial margin, pygopods short, subconical, each with erect setae. However, the known *Eleodes* larvae cannot yet be separated from other Amphidorini larvae due to a lack of specimens.

#### Subgenus *Blapylis* Horn, 1870

##### Eleodes (Blapylis) nigropilosus

(LeConte, 1851)

http://species-id.net/wiki/Eleodes_nigropilosus

Fig. 4A

###### Material examined.

Larval *Eleodes nigropilosus* specimens were reared from adults with the following collecting information: “USA: CA: San Diego Co. / Oceanside beach / 33.1865, -117.3778 / 14.May.2011, ADSmith”. A total of 29 eggs and larvae were reared and examined for this study, of which 34 survived to the 2nd instar or beyond. The following description is based on a detailed examination of three 8-11th instar specimens.

###### Description.

TL: 12–15.9 mm, HW: 1.0–1.1 mm, PL: 1.3–1.5 mm, PW: 1.0–1.2 mm.

**Head.** Prognathous or weakly declined; weakly dorsoventrally flattened; width nearly equal to prothorax; sides rounded; strongly constricted before occipital foramen; color light to dark tan, same or nearly the same as body segments; punctation minute, moderately dense, separated by 2–4 puncture diameters. Epicranial suture stem length approximately one-third head capsule length; frontal arms sinuate, not obscured by sculpturing. Frons faintly rugose. Epicranial plates weakly rugose dorsally; lateral portions moderately setose; ventral portion of each plate with row of four to five long setae along anterior margin near buccal cavity, not confluent with setae on lateral portions of plates, and a patch of short setae medially, forming a triangular pattern with its base near the anterior margin. Two stemmata present on each epicranial plate, pigmented spots often faded. Clypeus trapezoidal, not swollen, darker medially in basal half, minutely punctate, punctation moderately dense, separated by 2–4 puncture diameters. Labrum not swollen, sides rounded, basal half more darkly pigmented, medial setal row with six to seven erect setae subapical setal row with seven to eight erect setae, anterior margin straight to weakly emarginate. Epipharynx anterior setal row with six stout spiniform setae, anterolateral margins with micro-setation; six anterior sensory papillae present, arranged in two irregular diagonal rows; four subanterior sensory papillae present, arranged as transverse row subtended by two spinose setae; eight posterior sensory papillae present, arranged in an irregular cluster. Tormae asymmetric, left torma smaller. Ligula apex and subapical dorsal surface densely micro-setose, two long subapical setae present ventrally. Hypopharyngeal sclerome pentagonal, tricuspidate. Gula distinct, trapezoidal, widest in basal half, length subequal or greater than maximum width. Antenna three segmented, cylindrical, length of first segment subequal to second.

**Thorax.** Thoracic tergites light tan, prothoracic sternite anterior to legs medium brown, thoracic sternites posterior to prolegs light brown. Prothoracic tergum subquadrate, 1.5× length of meso- or metaterga; anterior transverse striated band present, darker than protergal disc; lateral margins with distinct granulated band, darker than protergal disc. Posterior transverse striated band present on all thoracic tergites, forming a gradient from darker brown anteriorly to lighter brown along posterior border. Meso- and metathoracic tergites wider than long, each with a faintly indicated sclerotized transverse line present on anterior fifth. Thoracic tergites sparsely setose on dorsal surfaces, lateral margins more densely setose. Mesothoracic spiracle simple, ovate, approximately 1.5× size of abdominal spiracles; reduced metathoracic spiracle visible, less than one-fourth size of mesothoracic spiracle. Legs. Prothoracic leg slightly longer, much thicker than meso- and metathoracic legs; prothoracic tarsungulus strongly sclerotized, sickle-shaped; prothoracic trochanter with two stout spines ventromedially; prothoracic femur with ventromedial row of three spines, dorsal surface with faintly indicated basal sclerotized band; prothoracic tibia with ventromedial row of three to four spines, dorsal surface slightly more sclerotized than ventral surface. Mesotibia with two ventromedial spines.

**Abdomen.** Abdominal tergites and sternites light tan with darker transverse striated bands present along posterior margins of segments I–VIII, forming near contiguous band around segments, bands dark along anterior edge, fading to segment color posteriorly. Abdominal sternite I sparsely clothed in long erect setae from anterior margin to near midline. Abdominal laterotergites with lateral margins distinctly pigmented. Abdominal segment IX (pygidium) triangular in dorsal view, gradually reflexed to apex, urogomphi absent, apex not forming a distinct tooth, moderately clothed in short and mid length erect setae, sclerotized uniformly throughout, lacking maculations; marginal row of 14–18 socketed spines present, arranged as single row around posterior two-thirds to one half of segment. Abdominal sternites I–VIII lacking longitudinal tomentose bands along lateral margins. Pygopods short, subconical, each with 9–12 erect setae.

###### Diagnosis.

*Eleodes nigropilosus* larvae can be separated from the other currently known *Eleodes* species by having the posterior pigmented band around the abdominal segments forming a color gradient from dark along anterior edge and fading to the color of the rest of the segment posteriorly.

#### Subgenus *Caverneleodes* Triplehorn, 1975

##### Eleodes (Caverneleodes) wheeleri

Eleodes Aalbu, Smith, & Triplehorn, 2012

http://species-id.net/wiki/Eleodes_wheeleri

Figs 2A , 4B

###### Material examined.

Larval E. wheeleri specimens were reared from adults with the following collecting information: “USA: Arizona: Gila Co. / Tonto Natural Bridge SP / N34.3214, W111.4569 / 11.IX.2010, ADSmith”. A total of 15 eggs and larvae were reared and examined for this study, with all surviving until the 2nd instar or beyond. The following description is based on a detailed examination of five 8-11th instar specimens.

###### Description.

Measurements: TL: 18.0–23.9 mm, PL: 1.6–2.1 mm, PW: 2.1–2.7 mm, HW: 1.8–2.3 mm.

**Head.** Prognathous or weakly declined; weakly dorsoventrally flattened; width nearly equal to prothorax; sides rounded; strongly constricted before occipital foramen; color light tan, same or nearly the same as body segments; punctation minute, dense, separated by 1–2 puncture diameters. Epicranial suture stem length approximately onethird head capsule length; frontal arms sinuate, not obscured by sculpturing. Frons weakly rugose. Epicranial plates weakly rugose dorsally; lateral portions sparsely setose; ventral portion of each plate with row of six or more long setae along anterior margin near buccal cavity confluent with setae on lateral portions of plates, and a patch of short setae medially, forming a triangular pattern with its base near the anterior margin. Two stemmata present on each epicranial plate, pigmented spots often faded. Clypeus trapezoidal, swollen, darker medially in basal half, minutely punctate, punctation moderately dense, separated by 2–4 puncture diameters. Labrum swollen, sides rounded, basal half more darkly pigmented, medial setal row with six to seven erect setae, subapical setal row with seven to eight erect setae, anterior margin straight to weakly emarginate. Epipharynx anterior setal row with six stout spiniform setae, anterolateral margins with micro-setation; four anterior sensory papillae present, arranged in two irregular longitudinal rows; four subanterior sensory papillae present arranged as a transverse row subtended by two spinose setae; eight posterior sensory papillae present, arranged in an irregular cluster. Tormae asymmetric, left torma smaller. Ligula apex lacking microsetae, two long subapical setae present ventrally, eight or more subapical setae present dorsally. Hypopharyngeal sclerome pentagonal, tricuspidate. Gula distinct, weakly trapezoidal, nearly rectangular. Antenna three segmented, cylindrical, first segment length subequal to second.

**Thorax.** Thoracic tergites light tan, prothoracic sternite anterior to legs light brown, thoracic sternites posterior to prolegs light tan to brown. Prothoracic tergum wider than long, 1.2× or more length of meso- or metaterga; anterior transverse striated band present, darker than protergal disc; lateral margins with very faint granulated band, nearly concolorous with protergal disc. Posterior transverse striated band present on all thoracic tergites, unicolorous brown. Meso- and metathoracic tergites wider than long, each with a faintly indicated sclerotized transverse line present on anterior fifth. Thoracic tergites sparsely setose on dorsal surfaces, lateral margins more densely setose. Mesothoracic spiracle simple, ovate, approximately 1.5× size of abdominal spiracles; reduced metathoracic spiracle visible, less than one-fourth size of mesothoracic spiracle. Legs. Prothoracic leg slightly longer, much thicker than meso- and metathoracic legs; prothoracic tarsungulus strongly sclerotized and sickle-shaped; prothoracic trochanter with two stout spines ventromedially; prothoracic femur with ventromedial row of four spines, dorsal surface with faintly indicated basal sclerotized band; prothoracic tibia with ventromedial row of five to six spines, dorsal surface slightly more sclerotized than ventral surface. Mesotibia with four to five ventromedial spines.

**Abdomen.** Abdominal tergites and sternites light tan with slightly darker transverse striated bands present along posterior margins of segments I–VIII, forming near contiguous unicolorous band around segments. Abdominal sternite I sparsely clothed in long erect setae along anterior margin. Abdominal laterotergites concolorous with tergites, lacking distinct pigmented margins. Abdominal segment IX (pygidium) triangular in dorsal view, gradually reflexed to apex, urogomphi absent, apex forming a small tooth, sparsely clothed in short and mid length erect setae, sclerotized uniformly throughout, lacking maculations; marginal row of 14–18 socketed spines present, arranged as single row around posterior two-thirds to one half of segment. Abdominal sternites I–VIII lacking longitudinal tomentose bands along lateral margins. Pygopods short, subconical, each with 11–15 erect setae.

###### Diagnosis.

Eleodes wheeleri larvae can be separated from the other currently known Eleodes species by the pentagonal hypopharyngeal sclerome, the lack of a distinct apical tooth on the pygidium, the presence of two long subapical ventral setae on the ligula with eight or more setae present dorsally, and the lateral margins of the protergum with a very faint granulated band, nearly concolorous with protergal disc.

###### Remarks.

Eleodes wheeleri was recently described (Aalbu et al. 2012) from Tonto Natural Bridge Caverns in Arizona and is known only from the type locality.

#### Subgenus *Eleodes* Eschscholtz, 1829

##### Eleodes (Eleodes) armatus

LeConte, 1851

http://species-id.net/wiki/Eleodes_armatus

Figs 2B , 4C , 9B , 11B

###### Material examined.

Larval *Eleodes armatus* specimens were reared from adults with the following collecting information: “USA: CA: Riverside Co. / Palm Desert, 38th Ave / off Washington St. / N33.7721, W116.3071 / 10.X.2010, ADSmith”; “USA: AZ: Maricopa Co. / Phoenix, E. Eugie Ave / & 7th St. N33°36.665’ / W112°03.849’, 418 m., / 25 May 2011, R.Dornburg.” A total of 1805 eggs and larvae were reared and examined for this study, with 128 persisting to the 2nd instar or later. The following description is based on a detailed examination of fifteen 8-11th instar specimens

###### Description.

TL: 21.0–35.0 mm, HW: 2.4–3.8 mm, PL: 2.4–3.4 mm, PW: 2.9–4.6 mm.

**Head.** Prognathous or weakly declined; weakly dorsoventrally flattened; width nearly equal to prothorax; sides rounded; strongly constricted before occipital foramen; color ferruginous, more heavily pigmented than body segments; punctation minute, dense, separated by 1–2 puncture diameters. Epicranial suture stem length approximately one-fourth head capsule length; frontal arms sinuate, not obscured by sculpturing. Frons weakly rugose. Epicranial plates weakly rugose dorsally; lateral portions moderately setose; ventral portion of each plate with row of six or more long setae along anterior margin near buccal cavity confluent with setae on lateral portions of plates, and a patch of short setae medially, forming a triangular pattern with its base near the anterior margin. Two stemmata present on each epicranial plate, pigmented spots often faded. Clypeus trapezoidal, swollen, darker medially in basal half, minutely punctate, punctation moderately dense, separated by 2–4 puncture diameters. Labrum swollen, sides rounded, basal half more darkly pigmented, medial setal row with seven to eight erect setae, subapical setal row with seven to eight erect setae, anterior margin straight to weakly emarginate. Epipharynx (Fig. 9B) anterior setal row with six stout spiniform setae, anterolateral margins with micro-setation; six anterior sensory papillae present, arranged in two irregular rows, each with two posterior papillae and one near the anterior margin; four subanterior sensory papillae present, arranged as a transverse row subtended by two spinose setae; eight posterior sensory papillae present, arranged in an irregular cluster. Tormae asymmetric, left torma smaller. Ligula apex lacking microsetae, two long subapical setae present ventrally, eight or more subapical setae present dorsally. Hypopharyngeal sclerome pentagonal, tricuspidate. Gula distinct, trapezoidal, widest in basal half, length less than maximum width. Antenna three segmented, cylindrical, first segment longer than second.

**Thorax.** Thoracic tergites light tan to ferruginous, prothoracic sternite anterior to legs ferruginous, thoracic sternites posterior to prolegs light brown. Prothoracic tergum wider than long, 1.2× or more length of meso-, metaterga; anterior transverse striated band present, darker than protergal disc; lateral margins with distinct granulated band, darker than protergal disc. Posterior transverse striated band present on all thoracic tergites, unicolorous brown. Meso- and metathoracic tergites wider than long, each with a heavily sclerotized transverse line present on anterior fifth. Thoracic tergites sparsely setose on dorsal surfaces, lateral margins more densely setose. Mesothoracic spiracle simple, ovate, approximately 1.5× size of abdominal spiracles; reduced metathoracic spiracle visible, less than one-fourth size of mesothoracic spiracle. Legs. Prothoracic leg slightly longer, much thicker than meso- and metathoracic legs; prothoracic tarsungulus strongly sclerotized, sickle-shaped; prothoracic trochanter with two stout spines ventromedially; prothoracic femur with ventromedial row of six to ten spines, dorsal surface with faintly indicated basal sclerotized band; prothoracic tibia with ventromedial row of eight to eleven spines or spinose setae, dorsal surface slightly more sclerotized than ventral surface. Mesotibia with five to seven ventromedial spines.

**Abdomen.** Abdominal tergites and sternites light tan to ferruginous, with slightly darker transverse striated bands present along posterior margins of segments I–VIII, forming near contiguous unicolorous band around segments. Abdominal sternite I moderately clothed in long erect setae from anterior margin to near midline. Abdominal laterotergites with lateral margins distinctly pigmented. Abdominal segment IX (pygidium) triangular in dorsal view, gradually reflexed to apex, urogomphi absent, apex forming a distinct tooth, sparsely clothed in short and mid length erect setae, sclerotized uniformly throughout, lacking maculations; marginal row of 22–24 socketed spines present, arranged as single row around posterior two-thirds to one half of segment. Abdominal sternites I–VIII lacking longitudinal tomentose bands along lateral margins. Pygopods short, subconical, each with 11–15 erect setae.

###### Diagnosis.

*Eleodes armatus* larvae can be separated from the other currently known *Eleodes* species by presence of an apical tooth on the pygidium and the absence of stout spiniform setae on the anterolateral margins of the epipharnyx.

##### Eleodes (Eleodes) caudiferus

LeConte, 1858

http://species-id.net/wiki/Eleodes_caudiferus

Figs 2C , 5A , 13A

###### Material examined.

Larval *Eleodes caudiferus* specimens were reared from adults with the following collecting information: “USA: Arizona: Navajo Co. / dunes ~4mi N Chilchinbito / off route 59, el. 1738m / N36.58143, W110.06973 / 26.August.2010, ADSmith”. A total of 85 eggs and larvae were reared and examined for this study, of which 53 survived untill the 2nd instar or later. The following description is based on a detailed examination of eleven 3-5th instar specimens.

###### Description.

TL: 7.8–12.8 mm, HW: 1.0–1.4 mm, PL: 1.0–1.8 mm, PW: 1.3–1.7 mm.

**Head.** Prognathous or weakly declined; weakly dorsoventrally flattened; width narrower than prothorax; sides rounded; strongly constricted before occipital foramen; color dark tan, same or nearly the same as on body segments; punctation minute, moderately dense, separated by 2–4 puncture diameters. Epicranial suture stem length approximately one-fourth to one-third head capsule length; frontal arms sinuate, not obscured by sculpturing. Frons rugose. Epicranial plates rugose dorsally; lateral portions densely setose; ventral portion of each plate with row of six or more long setae along anterior margin near buccal cavity confluent with setae on lateral portions of plates, and a patch of short setae medially, forming a triangular pattern with its base near the anterior margin. Two stemmata present on each epicranial plate, pigmented spots often faded. Clypeus trapezoidal, swollen, darker medially in basal half, minutely punctate, punctation moderately dense, separated by 2–4 puncture diameters. Labrum swollen, sides rounded, basal half more darkly pigmented, medial setal row with 10–14 erect setae, subapical setal row with 10–14 erect setae, anterior margin straight to weakly emarginate. Epipharynx anterior setal row with eight or more stout spiniform setae, anterolateral margins with micro-setation; six anterior sensory papillae present, arranged in two irregular rows; four subanterior sensory papillae present arranged as a transverse row subtended by two spinose setae; eight posterior sensory papillae present, arranged in an irregular cluster. Tormae symetrical or weakly asymmetric. Ligula apex densely microsetose, two long subapical setae present ventrally. Hypopharyngeal sclerome pentagonal, tricuspidate. Gula distinct, trapezoidal, widest in basal half, length less than maximum width. Antenna three segmented, cylindrical, first segment subequal to second.

**Thorax.** Thoracic tergites ferruginous, prothoracic sternite anterior to legs ferruginous, thoracic sternites posterior to prolegs light brown. Prothoracic tergum subquadrate, 1.5× length of meso- or metaterga; anterior transverse striated band present, darker than protergal disc; lateral margins with distinct granulated band, darker than protergal disc. Posterior transverse striated band present on all thoracic tergites, unicolorous brown. Meso- and metathoracic tergites wider than long, with sclerotized transverse line on anterior fifth absent, dense transverse band of short setae present near anterior margins of both tergites. Mesothoracic spiracle simple, ovate, approximately 1.5× size of abdominal spiracles; reduced metathoracic spiracle visible, less than one-fourth size of mesothoracic spiracle. Legs. Prothoracic leg slightly longer, much thicker than meso- and metathoracic legs; prothoracic tarsungulus strongly sclerotized, sickle-shaped; prothoracic trochanter with two stout spines ventromedially; prothoracic femur with ventromedial row of five to six spines, dorsal surface with faintly indicated basal sclerotized band; prothoracic tibia with ventromedial row of five to six spines or spinose setae, dorsal surface slightly more sclerotized than ventral surface. Mesotibia with row of three ventromedial spines.

**Abdomen.** Abdominal tergites and sternites light tan to ferruginous, with slightly darker transverse striated bands present along posterior margins of segments I–VIII, forming near contiguous unicolorous band around segments. Abdominal sternite I tomentose in anterior third, setae denser along near lateral margins. Abdominal laterotergites with lateral margins distinctly pigmented. Abdominal segment IX (pygidium) triangular in dorsal view, gradually reflexed to apex, urogomphi absent, apex attenuated and sclerotized, rarely forming a small tooth, sparsely clothed in short and mid length erect setae, sclerotized uniformly throughout, lacking maculations; marginal row of 28–38 socketed spines present, forming two or three irregular rows around posterior two-thirds to one half of segment, narrowing to single row around apex. Abdominal sternites I–VIII with longitudinal tomentose bands present along lateral margins. Pygopods short, subconical, each with 17–24 erect setae.

###### Diagnosis.

*Eleodes caudiferus* larvae can be separated from the other currently known *Eleodes* species by the presence of longitudinal tomentose bands along the lateral margins of abdominal sternites I–VIII.

##### Eleodes (Eleodes) hispilabris

(Say, 1824)

http://species-id.net/wiki/Eleodes_hispilabris

Figs 9C , 14A

###### Material examined.

Larval *Eleodes hispilabris* specimens were reared from adults with the following collecting information: “USA: TX: El Paso County / El Paso, sand dunes off / Hwy 180/Montana Ave. / N31.82327, W106.13234 / 21-22.VIII.2010, ADSmith”. A total of 46 eggs and larvae were reared and examined for this study, with 36 surviving until the 2nd instar or beyond. The following description is based on a detailed examination of five 8–11th instar specimens.

###### Description.

TL: 21.0–32.0 mm, PL: 2.6–3.2 mm, PW: 3.0–3.7 mm, HW: 2.4–3.1 mm.

**Head.** Prognathous or weakly declined; weakly dorsoventrally flattened; width narrower than prothorax; sides rounded; strongly constricted before occipital foramen; color ferruginous, more heavily pigmented than body segments; punctation minute, dense, separated by 1–2 puncture diameters. Epicranial suture stem length approximately one-fourth head capsule length; frontal arms sinuate, not obscured by sculpturing. Frons rugose. Epicranial plates rugose dorsally; lateral portions moderately setose; ventral portion of each plate with row of four to five long setae along anterior margin near buccal cavity, not confluent with setae on lateral portions of plates, with a patch of short setae medially, forming a triangular pattern with its base near the anterior margin. Two stemmata present on each epicranial plate, pigmented spots often faded. Clypeus trapezoidal, swollen, darker medially in basal half, minutely punctate, punctation dense, separated by 1–2 puncture diameters. Labrum swollen, sides rounded, basal half more darkly pigmented, medial setal row with six to seven erect setae, subapical setal row with 10–14 erect setae, anterior margin straight to weakly emarginate. Epipharynx (Fig. 9C) anterior setal row with eight or more stout spiniform setae, anterolateral margins with stout spinose setae; six anterior sensory papillae present, arranged in two irregular rows, each with two posterior papillae and one near the anterior margin; four subanterior sensory papillae present, arranged as a transverse row subtended by two spinose setae; seven to eight posterior sensory papillae present, arranged in an irregular cluster. Tormae strongly asymmetric, left torma larger. Ligula apex lacking microsetae, two long subapical setae present ventrally, eight or more subapical setae present dorsally. Hypopharyngeal sclerome pentagonal, tricuspidate. Gula distinct, trapezoidal, widest in basal half, length less than maximum width. Antenna three segmented, cylindrical, first segment longer than second.

**Thorax.** Thoracic tergites light tan, prothoracic sternite anterior to legs light tan to ferruginous, thoracic sternites posterior to prolegs light brown. Prothoracic tergum wider than long, 1.2× or more length of meso- or metaterga; anterior transverse striated band present, darker than protergal disc; lateral margins with distinct granulated band, darker than protergal disc. Posterior transverse striated band present on all thoracic tergites, unicolorous brown. Meso- and metathoracic tergites wider than long, each with a heavily sclerotized transverse line present on anterior fifth. Thoracic tergites sparsely setose on dorsal surfaces, lateral margins more densely setose. Mesothoracic spiracle simple, ovate, approximately 1.5× size of abdominal spiracles; reduced metathoracic spiracle visible, less than one-fourth size of mesothoracic spiracle. Legs. Prothoracic leg slightly longer, much thicker than meso- and metathoracic legs; prothoracic tarsungulus strongly sclerotized, sickle-shaped; prothoracic trochanter with one or two stout ventromedially spines; prothoracic femur with ventromedial row of six to ten spines, dorsal surface with faintly indicated basal sclerotized band; prothoracic tibia with ventromedial row of eight to eleven spines or spinose setae, dorsal surface slightly more sclerotized than ventral surface. Mesotibia with four to five ventromedial spines.

**Abdomen.** Abdominal tergites and sternites light tan, with slightly darker transverse striated bands present along posterior margins of segments I–VIII, forming near contiguous unicolorous band around segments. Abdominal sternite I sparsely clothed in long erect setae from anterior margin to near midline. Abdominal laterotergites with lateral margins distinctly pigmented. Abdominal segment IX (pygidium) triangular in dorsal view, gradually reflexed to apex, urogomphi absent, apex forming a distinct tooth, sparsely clothed in short and mid length erect setae, sclerotized uniformly throughout, lacking maculations; marginal row of 17–23 socketed spines present, arranged as single row around posterior two-thirds to one half of segment. Abdominal sternites I–VIII lacking longitudinal tomentose bands along lateral margins. Pygopods short, subconical, each with 9–12 erect setae.

###### Diagnosis.

*Eleodes hispilabris* larvae can be separated from the other currently known *Eleodes* species by the presence of an apical tooth on the pygidium, stout spiniform setae on the anterolateral margins of the epipharnyx, and a row of 6–10 ventromedial spines on the prothoracic femur.

##### Eleodes (Eleodes) tenuipes

Casey, 1890

http://species-id.net/wiki/Eleodes_tenuipes

###### Material examined.

Larval *Eleodes tenuipes* specimens were reared from adults with the following collecting information: “USA: TX: El Paso County / El Paso, sand dunes off / Hwy 180/Montana Ave. / N31.82327, W106.13234 / 21-22.VIII.2010, ADSmith”. Atotal of five eggs and larvae were reared and examined for this study. The following description is based on a detailed examination of one late instar specimen.

###### Description.

Measurements: TL: 39.0 mm, HW: 4.1 mm, PL: 4.0 mm, PW: 4.8 mm.

**Head.** Prognathous or weakly declined; weakly dorsoventrally flattened; width nearly equal to prothorax; sides rounded; strongly constricted before occipital foramen; color ferruginous, more heavily pigmented than body segments; punctation minute, dense, separated by 1–2 puncture diameters. Epicranial suture stem length approximately one-fourth head capsule length; frontal arms sinuate, not obscured by sculpturing. Frons rugose. Epicranial plates rugose dorsally; lateral portions moderately setose; ventral portion of each plate with row of six or more long setae along anterior margin near buccal cavity confluent with setae on lateral portions of plates, and a patch of short setae medially, forming a triangular pattern with its base near the anterior margin. Two stemmata present on each epicranial plate, pigmented spots often faded. Clypeus trapezoidal, swollen, darker medially in basal half, minutely punctate, punctation dense, separated by 1–2 puncture diameters. Labrum swollen, sides rounded, basal half more darkly pigmented, medial setal row with six to seven erect setae, subapical setal row with 10–14 erect setae, anterior margin straight to weakly emarginate. Epipharynx anterior setal row with eight or more stout spiniform setae, anterolateral margins with stout spinose setae; six anterior sensory papillae present, arranged in two irregular rows, each with two posterior papillae and one near the anterior margin; four subanterior sensory papillae present, arranged as a transverse row subtended by two spinose setae; eight posterior sensory papillae present, arranged in an irregular cluster. Tormae strongly asymmetric, left torma smaller. Ligula apex lacking microsetae, two long subapical setae present ventrally, eight or more subapical setae present dorsally. Hypopharyngeal sclerome pentagonal, tricuspidate. Gula distinct, trapezoidal, widest in basal half, length less than maximum width. Antenna three segmented, cylindrical, first segment longer than second.

**Thorax.** Thoracic tergites light tan, prothoracic sternite anterior to legs ferruginous, thoracic sternites posterior to prolegs light brown. Prothoracic tergum wider than long, 1.2× or more length of meso- or metaterga; anterior transverse striated band present, darker than protergal disc; lateral margins with distinct granulated band, darker than protergal disc. Posterior transverse striated band present on all thoracic tergites, unicolorous brown. Meso- and metathoracic tergites wider than long, each with a heavily sclerotized transverse line present on anterior fifth. Thoracic tergites sparsely setose on dorsal surfaces, lateral margins more densely setose. Mesothoracic spiracle simple, ovate, approximately 1.5× size of abdominal spiracles; reduced metathoracic spiracle visible, less than one-fourth size of mesothoracic spiracle. Legs. Prothoracic leg slightly longer, much thicker than meso- and metathoracic legs; prothoracic tarsungulus strongly sclerotized, sickle-shaped; prothoracic trochanter with one stout ventromedially spine; prothoracic femur with ventromedial row of 13–14 spines, dorsal surface with faintly indicated basal sclerotized band; prothoracic tibia with ventromedial row of eight to eleven spines or spinose setae, dorsal surface slightly more sclerotized than ventral surface. Mesotibia with five to seven ventromedial spines.

**Abdomen.** Abdominal tergites and sternites light tan, with slightly darker transverse striated bands present along posterior margins of segments I–VIII, forming near contiguous unicolorous band around segments. Abdominal sternite I sparsely clothed in long erect setae from anterior margin to near midline. Abdominal laterotergites with lateral margins distinctly pigmented. Abdominal segment IX (pygidium) triangular in dorsal view, gradually reflexed to apex, urogomphi absent, apex forming a distinct tooth, sparsely clothed in short and mid length erect setae, sclerotized uniformly throughout, lacking maculations; marginal row of 27 socketed spines present, arranged as single row around posterior two-thirds to one half of segment. Abdominal sternites I–VIII lacking longitudinal tomentose bands along lateral margins.

###### Diagnosis.

*Eleodes tenuipes* larvae can be separated from the other currently known *Eleodes* species by the presence of an apical tooth on the pygidium, stout spiniform setae on the anterolateral margins of the epipharnyx, and a row of 13–14 ventromedial spines on the prothoracic femur. It is further differentiated from *Eleodes hispilabris* by having a row of three ventromedial spines on the mesotarsus and having the ventral portion of the epicranial plates with a row of six or more long setae along anterior margin near buccal cavity, confluent with setae on lateral portions of plates.

###### Remarks.

Five eggs or early instar larvae were initially placed in a rearing chamber on 25 September 2010, though by the first sifting only one specimen was found. The last specimen thrived until 27 January 2011 when it was preserved for this study.

##### Eleodes (Eleodes) tribulus

Thomas, 2005

http://species-id.net/wiki/Eleodes_tribulus

Figs 2D , 5B , 9D

###### Material examined.

Larval *Eleodes tribulus* specimens were reared from adults with the following collecting information: “USA: AZ: Pinal Co. / I-10W Rest Area, mm183 / 33.029288, -111.771716 / 02 May 2011, ADSmith”. A total of 824 eggs and larvae were reared and examined for this study, of which 134 survived until the 2nd instar or later. The following description is based on a detailed examination of ten 8-11th instar specimens.

###### Description.

TL: 13.0–19.0 mm, HW: 1.5–2.2 mm, PL: 1.2–2.7 mm, PW: 1.3–2.7 mm.

**Head.** Prognathous or weakly declined; weakly dorsoventrally flattened; width nearly equal to prothorax; sides angular; strongly constricted before occipital foramen; color light tan to medium brown, more heavily pigmented than body segments; punctation minute, moderately dense, separated by 2–4 puncture diameters. Epicranial suture stem length approximately one-third head capsule length; frontal arms sinuate, not obscured by sculpturing. Frons rugose. Epicranial plates weakly rugose dorsally; lateral portions moderately setose; ventral portion of each plate with row of six or more long setae along anterior margin near buccal cavity confluent with setae on lateral portions of plates, and a patch of short setae medially, forming a triangular pattern with its base near the anterior margin. Two stemmata present on each epicranial plate, pigmented spots often faded. Clypeus trapezoidal, swollen, darker medially in basal half, minutely punctate, punctation moderately dense, separated by 2–4 puncture diameters. Labrum swollen, sides rounded, basal half more darkly pigmented, medial setal row with six to seven erect setae subapical setal row with six to seven erect setae, anterior margin straight to weakly emarginate. Epipharynx (Fig. 9D) anterior setal row with six stout spiniform setae, anterolateral margins with micro-setation; five to six anterior sensory papillae present, arranged in two irregular longitudinal rows or an irregular cluster; four subanterior sensory papillae present, arranged as a transverse row subtended by two spinose setae; seven to eight posterior sensory papillae present, arranged in an irregular cluster. Tormae asymmetric, left torma larger. Ligula apex and subapical dorsal surface densely micro-setose, two long subapical setae present ventrally. Hypopharyngeal sclerome pentagonal, tricuspidate. Gula distinct, trapezoidal, widest in basal half, length subequal or greater than maximum width. Antenna three segmented, cylindrical, first segment length subequal to second.

**Thorax.** Thoracic tergites light tan, prothoracic sternite anterior to legs medium brown, thoracic sternites posterior to prolegs light brown. Prothoracic tergum subquadrate, 1.5× length of meso- or metaterga; lateral margins with distinct granulated band, darker than protergal disc; anterior transverse striated band present, darker than tergal disc. Posterior transverse striated band present on all thoracic tergites, unicolorous brown. Meso- and metathoracic tergites wider than long, each with a faintly indicated sclerotized transverse line present on anterior fifth. Thoracic tergites sparsely setose on dorsal surfaces, lateral margins more densely setose. Mesothoracic spiracle simple, ovate, approximately 1.5× size of abdominal spiracles; reduced metathoracic spiracle visible, less than one-fourth size of mesothoracic spiracle. Legs. Prothoracic leg slightly longer, much thicker than meso- and metathoracic legs; prothoracic tarsungulus strongly sclerotized, sickle-shaped; prothoracic trochanter with two stout spines ventromedially; prothoracic femur with ventromedial row of two spines and three to five longer setae, dorsal surface with faintly indicated basal sclerotized band; prothoracic tibia with ventromedial row of three to four spines, dorsal surface slightly more sclerotized than ventral surface. Mesotibia with three ventromedial spines.

**Abdomen.** Abdominal tergites and sternites light tan with darker transverse striated bands present along posterior margins of segments I–VIII, forming near contiguous unicolorous band around segments. Abdominal sternite I moderately clothed in long erect setae from anterior margin to near midline. Abdominal laterotergites with lateral margins distinctly pigmented. Abdominal segment IX (pygidium) triangular in dorsal view, gradually reflexed to apex, urogomphi absent, apex not forming a distinct tooth, moderately clothed in short and mid length erect setae, sclerotized uniformly throughout, lacking maculations; marginal row of 8–14 socketed spines present, arranged as single row around posterior two-thirds to one half of segment. Abdominal sternites I–VIII lacking longitudinal tomentose bands along lateral margins. Pygopods short, subconical, each with 11–15 erect setae.

###### Diagnosis.

*Eleodes tribulus* larvae can be separated from the other currently known *Eleodes* species based on the pentagonal hypopharyngeal sclerome, lack of a caudal tooth on the pygidium, presence of 8–14 marginal spines on the pygidium, and the angular, nearly straight sides of the head capsule.

#### Subgenus *Litheleodes* Blaisdell, 1909

##### Eleodes (Litheleodes) extricatus

(Say, 1823)

http://species-id.net/wiki/Eleodes_extricatus

Figs 3A , 5C , 7B , 10A , 12B , 13B

###### Material examined.

Larval *Eleodes extricatus* specimens were reared from adults with the following collecting information: “USA: TX: El Paso County / El Paso, sand dunes off / Hwy 180/Montana Ave. / N31.82327, W106.13234 / 21–22.VIII.2010, ADSmith”, “USA: Arizona: Navajo Co. / dunes ~4mi N Chilchinbito / off route 59, el. 1738m / N36.58143, W110.06973 / 26.August.2010, ADSmith”, “USA: AZ: Graham Co. / Pinaleño Mnts, Hospital Flat Camp / N32°39’58.0”, W109°52’30.9” / el.9000’ 22.Aug.2010 / ADSmith”, “USA: Arizona: Gila County / E. Verde River off NF-272 / N34.303, W111.3496 / 27.August.2010, ADSmith”. Approximately 219 eggs and larvae were reared and examined for this study, with 150 surviving until the second instar or beyond. The following description is based on a detailed examination of thirteen 8–11th instar specimens.

###### Description.

Measurements: TL: 15.4–33.3 mm, PL: 2.4–3.8 mm, PW: 2.2–3.8 mm, HW: 2.0–3.0 mm.

**Head.** Prognathous or weakly declined; weakly dorsoventrally flattened; width nearly equal to prothorax; sides rounded; strongly constricted before occipital foramen; color light tan, same or nearly the same as body segments; punctation minute, dense, separated by 1–2 puncture diameters. Epicranial suture stem length approximately one-third head capsule length; frontal arms sinuate, not obscured by sculpturing. Frons faintly rugose. Epicranial plates faintly rugose dorsally; lateral portions moderately setose; ventral portion of each plate with row of six or more long setae along anterior margin near buccal cavity confluent with setae on lateral portions of plates and a patch of short setae medially, forming a triangular pattern with its base near the anterior margin. Two stemmata present on each epicranial plate, pigmented spots often faded. Clypeus trapezoidal, swollen or not, unicolorous, minutely punctate, punctation dense, separated by 1–2 puncture diameters. Labrum swollen, sides rounded, basal half more darkly pigmented, medial setal row with six to seven erect setae, subapical setal row with six to seven erect setae, anterior margin straight to weakly emarginate. Epipharynx (Fig. 10A) anterior setal row with six stout spiniform setae, anterolateral margins with micro-setation; six anterior sensory papillae present, arranged in two irregular rows; four subanterior sensory papillae present, arranged as a transverse row subtended by two spinose setae; eight posterior sensory papillae present, arranged in an irregular cluster. Tormae symmetrical or weakly asymmetrical with left torma smaller. Ligula apex densely microsetose, two long subapical setae present ventrally. Hypopharyngeal sclerome pentagonal, tricuspidate. Gula distinct, trapezoidal, widest in basal half, length less than maximum width. Antenna three segmented, cylindrical, first segment longer than second.

**Thorax.** Thoracic tergites light tan, prothoracic sternite anterior to legs ferruginous, thoracic sternites posterior to prolegs light brown. Prothoracic tergum subquadrate, 1.5× length of meso- or metaterga; anterior transverse striated band present, darker than protergal disc; lateral margins with distinct granulated band, darker than protergal disc. Posterior transverse striated band present on all thoracic tergites, unicolorous brown. Meso- and metathoracic tergites wider than long, each with a heavily sclerotized transverse line present on anterior fifth. Thoracic tergites sparsely setose on dorsal surfaces, lateral margins more densely setose. Mesothoracic spiracle simple, ovate, approximately 1.5× size of abdominal spiracles; reduced metathoracic spiracle visible, less than one-fourth size of mesothoracic spiracle. Legs. Prothoracic leg slightly longer, much thicker than meso- and metathoracic legs; prothoracic tarsungulus strongly sclerotized, sickle-shaped; prothoracic trochanter with two stout ventromedially spines; prothoracic femur with ventromedial row of two spines and three to five longer setae, dorsal surface with faintly indicated basal sclerotized band; prothoracic tibia with ventromedial row of three to four spines or spinose setae, dorsal surface slightly more sclerotized than ventral surface. Mesotibia with four to five ventromedial spines.

**Abdomen.** Abdominal tergites and sternites light tan, with slightly darker transverse striated bands present along posterior margins of segments I–VIII, forming near contiguous unicolorous band around segments. Abdominal sternite I sparsely clothed in long erect setae from anterior margin to near midline. Abdominal laterotergites with lateral margins distinctly pigmented. Abdominal segment IX (pygidium) triangular in dorsal view, gradually reflexed to apex, urogomphi absent, apex lacking a distinct tooth, sparsely clothed in short and mid length erect setae, sclerotized uniformly throughout, lacking maculations; marginal row of 17–23 socketed spines present, arranged as single row around posterior two-thirds to one half of segment. Abdominal sternites I–VIII lacking longitudinal tomentose bands along lateral margins. Pygopods short, subconical, each with 11–15 erect setae.

###### Diagnosis.

*Eleodes extricatus* larvae can be separated from the other currently known *Eleodes* species based on the pentagonal hypopharyngeal sclerome, small or absent apical tooth on the pygidium, lateral margins of prothoracic tergum with a distinct granulated band, and having antennal segment I longer than antennal segment II.

###### Remarks.

*Eleodes extricatus* is a widespread species found on dunes and at high elevations. Specimens from Arizona and Texas showed no population differences in the larval stage. Adults varied in the presence or prominence of muricate tubercles on the elytra.

#### Subgenus *Melaneleodes* Blaisdell, 1909

##### Eleodes (Melaneleodes) anthracinus

Blaisdell, 1909

http://species-id.net/wiki/Eleodes_anthracinus

Figs 3B , 6A , 7A , 8

###### Material examined.

Larval *Eleodes anthracinus* specimens were reared from adults with the following collecting information: “USA: AZ: Maricopa Co. / Eugie Ave & 7th St. / 25 Oct. 2011, R. Dornburg.” A total of 28 eggs and larvae were reared and examined for this study, of which all survived until the 3rd instar or later. The following description is based on a detailed examination of four 8–11th instar specimens.

###### Description.

TL: 23.8–28.1 mm, HW: 2.3–2.4 mm, PL: 2.0–2.4 mm, PW: 2.5–2.8 mm.

**Head.** Prognathous or weakly declined; weakly dorsoventrally flattened; width nearly equal to prothorax; sides rounded; strongly constricted before occipital foramen; color medium brown to brown-grey, nearly as on body segments; minute punctation moderately dense dorsally. Epicranial stem approximately one-third head capsule length; frontal arms U-shaped, not obscured by sculpturing. Frons and dorsal portion of epicranial plates faintly rugose; lacking non-primary setae. Lateral portions of epicranial plates moderately setose; setae golden, erect, length equal to or longer than antennal segment 2; ventral portions of epicranial plates with a row of four long setae along anterior margin near buccal cavity with a patch of short setae medially forming a triangular pattern with its base near the anterior margin; two stemmata present on each plate, pigmented spots often faded. Clypeus trapezoidal; not swollen, moderately punctate, darker medially in basal half. Labrum not swollen, basal half more darkly pigmented; sides rounded; two transverse rows of seven to eight erect setae present medially and subapically; anterior margin straight. Epipharynx (Fig. 3) anterior setal row with six stout spiniform setae, anterolateral margins with micro-setation; six anterior sensory papillae present, arranged in two irregular diagonal rows; four subanterior sensory papillae present, arranged as a transverse row subtended by two spinose setae; eight posterior sensory papillae present, arranged in two irregular rows.Tormae asymmetrical, left torma smaller. Ligula with four long setae near apex. Hypopharyngeal sclerome trapezoidal. Gula distinct, trapezoidal, widest in basal half. Antenna three segmented, cylindrical; first segment longer than second.

**Thorax.** Grey-brown to medium brown dorsally and anterior to legs on prothoracic sternite, tan on rest of sternites; lighter transverse striated band present along anterior fourth of prothoracic tergum; thin darkly sclerotized transverse line present on anterior fifth of meso- and metathoracic tergites; striated bands present along posterior 5th of all thoracic tergites, color forming a gradient from darker brown anteriorly to lighter brown along posterior border. Eight evenly arranged setae present on dorsal surface of each thoracic terga, lateral margins more densely setose. Prothoracic tergum subquadrate, 1.5× length of meso- or metaterga; lateral margins lacking pigmented band. Meso- and metaterga wider than long, lacking pigmented bands along lateral margins; mesothoracic spiracle simple, ovate, approximately 1.5× size of abdominal spiracle; reduced metathoracic spiracle visible, less than one-fourth size of mesothoracic spiracle. Prothoracic leg slightly longer, much thicker than meso- and metathoracic legs; prothoracic tarsungulus strongly sclerotized, sickle-shaped; trochanter with row of two stout spines and two longer setae ventromedially, tibia with ventromedial row of two spines and four to five longer setae, tarsus with ventromedial row of four spines. Dorsal surface of protibia (at rest) with faintly indicated basal sclerotized band; dorsal surface of protarsus slightly more sclerotized than ventral surface.

**Abdomen.** Tergites grey-brown to medium brown dorsally, lightening towards lateral margins, sternites light to dark tan; transverse striated bands not visible on abdominal sternites, barely visible on posterior 5th of terga I–VIII, nearly concolorous with rest of tergites. Abdominal sternite I sparsely clothed in long erect setae from anterior margin to near midline, abdominal segments II–VIII each with two sparse transverse bands of long erect setae, posterior margin of segment 8 denser setal band. Abdominal laterotergites concolorous with tergites, lacking distinct pigmented margins. Abdominal segment IX (pygidium) triangular in dorsal view, gradually reflexed to apex, sparsely clothed in short and mid length erect setae, dorsally more sclerotized in apical two-thirds with faint maculations; marginal row of 14–18 socketed spines present apical half, apex not forming distinct sclerotized projection. Pygopods short, subconical, each with 11–15 erect spines.

**Variation.** Little variation was observed between specimens beyond the number of spines on the legs and pygidium, and the overall degree of sclerotization.

###### Diagnosis.

*Eleodes anthracinus* larvae can be separated from most currently known *Eleodes* species based on their darker dorsal coloration on all segments, the absence of pigmented bands along the lateral margins of the thoracic terga, and the lack of a distinct sclerotized tooth at the apex of the pygidium. They can be distinguished from *Eleodes carbonarius* larvae by their lighter ventral segments and lack of distinct posterior pigmented bands on the abdominal terga. Larvae of *Eleodes tricostatus* (Say), another species in the subgenus *Melaneleodes*, are mentioned as being “nearly black” by McColloch (1918). However, no other diagnostic characters are mentioned that would separate them from the other *Eleodes anthracinus* or *Eleodes carbonarius*.

##### Eleodes (Melaneleodes) carbonarius knausii

Blaisdell

Figs 3C , 6B , 9A , 11A , 12A

###### Material examined.

Larval *Eleodes carbonarius* specimens were reared from adults with the following collecting information: “USA: CO: Montezuma Co. / Ute RA off Hwy 160 / 37.3535, -108.44385 / 05 Jun 2011, ADSmith”. A total of 129 eggs and larvae were reared and examined for this study, with 45 surviving until the 2nd instar or later. The following description is based on a detailed examination of five 8–11th instar specimens.

###### Description.

TL: 15.5–26 mm, HW: 2.3–3.0 mm, PL: 1.9–2.5 mm, PW: 3.0–3.5 mm.

**Head.** Prognathous, weakly flattened, narrower than prothorax; sides rounded, strongly constricted before occipital foramen; color ferruginous to dark brown, nearly as on body segments; minute punctation moderately dense dorsally. Epicranial stem approximately one-third head capsule length; frontal arms U-shaped, not obscured by sculpturing. Frons and dorsal portion of epicranial plates faintly rugose; lacking non-primary setae. Lateral portions of epicranial plates moderately setose; setae golden, erect, length equal to or longer than antennal segment 2; ventral portions of epicranial plates with a row of four to five long setae along anterior margin near buccal cavity and a patch of short setae medially forming a triangular pattern with its base near the anterior margin; two stemmata present on each plate, pigmented spots often faded. Clypeus trapezoidal; not swollen, moderately punctate, darker medially in basal half. Labrum not swollen, basal half more darkly pigmented; sides rounded; two transverse rows of six to seven erect setae present medially and subapically; anterior margin straight to weakly emarginate. Epipharynx (Fig. 9A) anterior setal row with six stout spiniform setae, anterolateral margins with micro-setation; six anterior sensory papillae present, arranged in two irregular diagonal rows; four subanterior sensory papillae present, arranged as a transverse row subtended by two spinose setae; eight posterior sensory papillae present, arranged in an irregular cluster. Tormae asymmetrical, left torma larger. Hypopharyngeal sclerome trapezoidal. Ligula with four long setae near apex. Gula distinct, trapezoidal, widest in basal half. Antenna three segmented, cylindrical; first segment longer than second.

**Thorax.** Dark brown to ferruginous dorsally and anterior to legs on prothoracic sternite, lighter brown on rest of sternites; distinct longitudinally striated band present along anterior fourth of prothoracic tergum; thin darkly sclerotized transverse line present on anterior fifth of meso- and metathoracic tergites; striated bands present along posterior 6th of all thoracic tergites, darker than rest of surface. Eight evenly arranged setae present on dorsal surface of each thoracic terga, lateral margins more densely setose. Prothoracic tergum wider than long, 1.5× length of meso- or metaterga; lateral margins lacking pigmented band. Meso- and metaterga wider than long, lacking pigmented bands along lateral margins; mesothoracic spiracle simple, ovate, approximately 1.5× size of abdominal spiracle; reduced metathoracic spiracle visible, less than one-fourth size of mesothoracic spiracle. Prothoracic leg slightly longer, much thicker than meso- and metathoracic legs; prothoracic tarsungulus strongly sclerotized, sickle-shaped; trochanter with two stout spines ventromedially, tibia with ventromedial row of three to four spines and four to five longer setae, tarsus with ventromedial row of five spines. Dorsal surface of protibia (at rest) with basal sclerotized band; dorsal surface of protarsus more sclerotized than ventral surface.

**Abdomen.** Tergites dark brown to ferruginous, concolorous or lightly lighter than tergites; longitudinally striated bands not visible on abdominal sternites, distinct on posterior 5th of terga 1–8. Abdominal sternite I sparsely clothed in long erect setae from anterior margin to near midline, abdominal segments 2–8 each with two sparse transverse bands of long erect setae, posterior margin of segment 8 denser setal band. Abdominal laterotergites concolorous with tergites, lacking distinct pigmented margins. Abdominal segment IX (pygidium) triangular in dorsal view, gradually reflexed to apex, sparsely clothed in short and mid length erect setae, apical two-thirds with faint maculations; marginal row of 18–20 socketed spines present apical half, apex not forming distinct sclerotized projection. Pygopods short, subconical, each with 9–12 erect spines.

**Variation.** Little variation was observed between specimens beyond the number of spines on the legs and pygidium, and the overall degree of sclerotization.

###### Diagnosis.

*Eleodes carbonarius* larvae can be separated from most currently known *Eleodes* species their darker dorsal coloration on all segments, the absence of pigmented bands along the lateral margins of the thoracic terga, and the lack of a distinct sclerotized tooth at the apex of the pygidium. They can be further distinguished from *Eleodes anthracinus* larvae as outlined in that species diagnosis.

###### Remarks.

*Eleodes carbonarius* adult morphology is notoriously variable across the species range and even within populations. Nine subspecies are currently recognized (Triplehorn and Thomas 2011). The specimens examined were all reared from a few females of *Eleodes carbonarius knausi* Blaisdell collected at a single locality. Larval characters showed little variation; however, this may change as more specimens are reared from other localities and subspecies.

#### Subgenus *Promus* LeConte, 1862

##### Eleodes (Promus) goryi

Solier, 1848

http://species-id.net/wiki/Eleodes_goryi

Figs 10B , 11C

###### Material examined.

Larval *Eleodes goryi* specimens were reared from adults with the following collecting information: “USA: TX: Hidalgo County / Bentsen-Rio Grande Valley / State Park, fm2062 Mission / N26°10.37’, W098°22.93’ / 02.Sept.2011, Aaron Smith”. A total of 460 eggs and larvae were reared and examined for this study, with 25 surviving until the 2nd instar or beyond. The following description is based on a detailed examination of three 8–11th instar specimens.

###### Description.

TL: 25.0–25.4 mm, HW: 2.0–2.1 mm, PL: 2.0–2.1 mm, PW: 2.2–2.4 mm.

**Head.** Prognathous or weakly declined; weakly dorsoventrally flattened; width nearly equal to prothorax; sides rounded; strongly constricted before occipital foramen; color ferruginous to dark brown, more heavily pigmented than body segments; punctation minute, moderately dense, separated by 2–4 puncture diameters. Epicranial suture stem length approximately one-third head capsule length; frontal arms U-shaped, not obscured by sculpturing. Frons faintly rugose. Epicranial plates faintly rugose dorsally; lateral portions moderately setose; ventral portion of each plate with row of six or more long setae along anterior margin near buccal cavity confluent with setae on lateral portions of plates, and a patch of short setae medially, forming a triangular pattern with its base near the anterior margin. Two stemmata present on each epicranial plate, pigmented spots often faded. Clypeus trapezoidal, swollen, darker medially in basal half, minutely punctate, punctation moderately dense, separated by 2–4 puncture diameters. Labrum swollen, sides rounded, basal half more darkly pigmented, medial setal row with six to seven erect setae, subapical setal row with six to seven erect setae, anterior margin straight to weakly emarginate. Epipharynx (Fig. 10B) anterior setal row with six stout spiniform setae, anterolateral margins with micro-setation; six anterior sensory papillae present, arranged in two irregular rows; four subanterior sensory papillae present, arranged as a transverse row subtended by two spinose setae; eight posterior sensory papillae present, arranged in an irregular cluster. Tormae strongly asymetrical with left torma larger. Ligula apex densely microsetose, two long subapical setae present ventrally. Hypopharyngeal sclerome pentagonal, tricuspidate. Gula distinct, trapezoidal, widest in basal half, length subequal or greater than maximum width. Antenna three segmented, cylindrical, first segment subequal to second.

**Thorax.** Thoracic tergites light tan, prothoracic sternite anterior to legs ferruginous to medium brown, thoracic sternites posterior to prolegs medium brown. Prothoracic tergum wider than long, 1.2× or more length of meso- or metaterga; anterior transverse striated band present, darker than protergal disc; lateral margins with distinct granulated band, darker than protergal disc. Posterior transverse striated band present on all thoracic tergites, unicolorous brown. Meso- and metathoracic tergites wider than long, each with a heavily sclerotized transverse line present on anterior fifth. Thoracic tergites sparsely setose on dorsal surfaces, lateral margins more densely setose. Mesothoracic spiracle simple, ovate, approximately 1.5× size of abdominal spiracles; reduced metathoracic spiracle visible, less than one-fourth size of mesothoracic spiracle. Legs. Prothoracic leg slightly longer, much thicker than meso- and metathoracic legs; prothoracic tarsungulus strongly sclerotized, sickle-shaped; prothoracic trochanter with two stout ventromedially spines; prothoracic femur with ventromedial row of two spines and three to five longer setae, dorsal surface with faintly indicated basal sclerotized band; prothoracic tibia with ventromedial row of three to four spines or spinose setae, dorsal surface slightly more sclerotized than ventral surface. Mesotibia with three ventromedial spines.

**Abdomen.** Abdominal tergites and sternites 1–7 light tan, with slightly darker transverse striated bands present along posterior margins of segments I–VIII, forming near contiguous unicolorous band around segments. Abdominal tergite 8 more darkly pigmented than preceding segments. Abdominal sternite I moderately clothed in long erect setae from anterior margin to near midline. Abdominal laterotergites with lateral margins distinctly pigmented. Abdominal segment IX (pygidium) triangular in dorsal view, gradually reflexed to apex, urogomphi absent, apex lacking a distinct tooth, moderately clothed in short and mid length erect setae, dorsally more sclerotized in apical two-thirds with faint maculations; marginal row of 18–20 socketed spines present, arranged as single row around posterior two-thirds to one half of segment. Abdominal sternites I–VIII lacking longitudinal tomentose bands along lateral margins. Pygopods short, subconical, each with 11–15 erect setae.

###### Diagnosis.

*Eleodes goryi* larvae can be separated from the other currently known *Eleodes* species based on the darkly pigmented eighth and ninth abdominal tergites. It is further distinguished by the pentagonal hypopharyngeal sclerome, lack of a caudal tooth on the pygidium, and the presence of 3–4 ventromedial spines on the protibia.

##### Eleodes (Promus) subnitens

LeConte, 1851

http://species-id.net/wiki/Eleodes_subnitens

Figs 7C , 10C

###### Material examined.

Larval *Eleodes subnitens* specimens were reared from adults with the following collecting information: “USA: Arizona: Gila Co. / Tonto Natural Bridge SP / N34.3214, W111.4569 / 11.IX.2010, ADSmith”. A total of 7 eggs and larvae were reared and examined for this study, of which four survived until the 2nd instar or later. The following description is based on a detailed examination of two 8–11th instar specimens.

###### Description.

TL: 23.1–30.8 mm, HW: 2.0–3.0 mm, PL: 2.0–2.9 mm, PW: 2.2–3.1 mm.

**Head.** Prognathous or weakly declined; weakly dorsoventrally flattened; width nearly equal to prothorax; sides rounded; strongly constricted before occipital foramen; color ferruginous, more heavily pigmented than body segments; punctation minute, moderately dense, separated by 2–4 puncture diameters. Epicranial suture stem length approximately one-third head capsule length; frontal arms sinuate, not obscured by sculpturing. Frons faintly rugose. Epicranial plates faintly rugose dorsally; lateral portions moderately setose; ventral portion of each plate with row of six or more long setae along anterior margin near buccal cavity confluent with setae on lateral portions of plates and a patch of short setae medially, forming a triangular pattern with its base near the anterior margin. Two stemmata present on each epicranial plate, pigmented spots often faded. Clypeus trapezoidal, swollen, darker in apical half, minutely punctate, punctation moderately dense, separated by 2–4 puncture diameters. Labrum swollen, sides rounded, basal half more darkly pigmented, medial setal row with six to seven erect setae, subapical setal row with seven to eight erect setae, anterior margin straight to weakly emarginate. Epipharynx (Fig. 10C) anterior setal row with six stout spiniform setae, anterolateral margins with micro-setation; six anterior sensory papillae present, arranged in two irregular rows; four subanterior sensory papillae present, arranged as a transverse row subtended by two spinose setae; eight posterior sensory papillae present, arranged in an irregular cluster. Tormae asymetrical with left torma smaller. Ligula apex densely microsetose, two long subapical setae present ventrally. Hypopharyngeal sclerome pentagonal, tricuspidate. Gula distinct, trapezoidal, widest in basal half, length subequal or greater than maximum width. Antenna three segmented, cylindrical, first segment subequal to second.

**Thorax.** Thoracic tergites light tan, prothoracic sternite anterior to legs ferruginous, thoracic sternites posterior to prolegs light brown. Prothoracic tergum wider than long, 1.2× or more length of meso- or metaterga; anterior transverse striated band present, darker than protergal disc; lateral margins with distinct granulated band, darker than protergal disc. Posterior transverse striated band present on all thoracic tergites, unicolorous brown. Meso- and metathoracic tergites wider than long, each with a heavily sclerotized transverse line present on anterior fifth. Thoracic tergites sparsely setose on dorsal surfaces, lateral margins more densely setose. Mesothoracic spiracle simple, ovate, approximately 1.5× size of abdominal spiracles; reduced metathoracic spiracle visible, less than one-fourth size of mesothoracic spiracle. Legs. Prothoracic leg slightly longer, much thicker than meso- and metathoracic legs; prothoracic tarsungulus strongly sclerotized, sickle-shaped; prothoracic trochanter with two stout ventromedially spines; prothoracic femur with ventromedial row of two spines and three to five longer setae, dorsal surface with faintly indicated basal sclerotized band; prothoracic tibia with ventromedial row of five to six spines or spinose setae, dorsal surface slightly more sclerotized than ventral surface. Mesotibia with four to five ventromedial spines.

**Abdomen.** Abdominal tergites and sternites I–VIII light tan, with slightly darker transverse striated bands present along posterior margins8, forming near contiguous unicolorous band around segments. Abdominal sternite I moderately clothed in long erect setae to posterior pigmented band. Abdominal laterotergites with lateral margins distinctly pigmented. Abdominal segment IX (pygidium) triangular in dorsal view, gradually reflexed to apex, urogomphi absent, apex lacking a distinct tooth, moderately clothed in short and mid length erect setae, dorsally sclerotization uniform throughout, lacking maculations; marginal row of 18–20 socketed spines present, arranged as single row around posterior two-thirds to one half of segment. Abdominal sternites 1–8 lacking longitudinal tomentose bands along lateral margins. Pygopods short, subconical, each with 17–24 erect setae.

###### Diagnosis.

*Eleodes subnitens* larvae can be separated from the other currently known *Eleodes* species by the pentagonal hypopharyngeal sclerome, prothoracic tergum wider than long, 8th and 9th abdominal tergites not darker than proceeding segments, lack of a caudal tooth on the pygidium, and the presence of 5–6 ventromedial spines on the protibia.

#### Subgenus *Tricheleodes* Blaisdell, 1909

##### Eleodes (Tricheleodes) pilosus

Horn, 1870

http://species-id.net/wiki/Eleodes_pilosus

Figs 3D , 6C , 10D , 14B

###### Material examined.

Larval *Eleodes pilosus* specimens were reared from adults with the following collecting information: “NEVADA: Washoe Co. / N39°16.427’, W119°47.070’ / November 14, 2011 / P.Skelley, sifting lakeside dunes”. A total of 208 eggs and larvae were reared and examined for this study, of which 94 survived until the 2nd instar or later. The following description is based on a detailed examination of nine 8–11th instar specimens.

###### Description.

TL: 14.2–26.0 mm, PW: 1.7–3.3 mm, PL: 1.4–3.4 mm, HW: 1.6–2.6 mm.

**Head.** Prognathous, weakly flattened, narrower than prothorax; sides rounded, strongly constricted before occipital foramen; color nearly as in body segments. Epicranial stem short, one-fourth head capsule length; frontal arms U-shaped, partially obscured by sculpturing. Frons and dorsal portion of epicranial plates rugose; sparsely setose; densely punctate, punctures minute, lacking setae. Ventrolateral portions of epicranial plates densely setose; setae golden, erect, most longer than antennal segment 2, interspersed with shorter setae; two stemmata present on each plate, pigmented spots often faded. Clypeus trapezoidal; swollen, weakly transversely raised medially; moderately punctate, rugose in basal half. Labrum slightly swollen, basal fourth darkly pigmented; sides rounded, minutely tomentose; two transverse rows of six to eight erect setae present medially and subapically; anterior margin straight. Epipharynx (Fig. 10D) anterior setal row with six stout spiniform setae, anterolateral margins with micro-setation; seven anterior sensory papillae present, arranged in two irregular longitudinal rows; four subanterior sensory papillae present, arranged as a transverse row subtended by two spinose setae; seven posterior sensory papillae present, arranged in an irregular cluster. Tormae asymmetrical, left side torma smaller with or without a small spine near emergent edge. Hypopharyngeal sclerome pentagonal, tricuspidate. Gula distinct, trapezoidal, widest in basal half. Antenna three segmented, cylindrical; first segment longer than second.

**Thorax.** Light to dark tan, darker longitudinally striated bands present on anterior margin of prothoracic tergum and posterior 5th of all thoracic tergites. Sparsely setose along dorsal margins of terga near striated bands, lateral margins more densely setose. Prothoracic tergum subquadrate, 1.5× length of meso- or metaterga; lateral margins with pigmented band along entire length. Mesothoracic spiracle simple, ovate, approximately 1.5× size of abdominal spiracle; reduced metathoracic spiracle visible, less than one-fourth size of mesothoracic spiracle. Prothoracic leg slightly longer, much thicker than meso- and metathoracic legs; tarsungulus strongly sclerotized, sickle-shaped; trochanter with two stout spines ventromedially, tibia and tarsus each with a ventromedial row of four to seven spines, number of spines often differing between prolegs. Dorsal surface of protibia (at rest) with basal sclerotized band; dorsal surface of protarsus sclerotized.

**Abdomen.** Light to dark tan, darker longitudinally striated bands present on posterior 5th of segments I–VIII. Abdominal sternite I moderately clothed in long erect setae, sparser medially, extending to posterior pigmented band, abdominal tergite I and segments II–VIII sparsely clothed in short to mid length setae. Lateral margins of abdominal laterotergites I–VIII darkly pigmented, ventral margin with two pigmented bands. Abdominal segment IX (pygidium) triangular in dorsal view, moderately clothed in long erect setae; marginal row of 10–20 socketed spines present apical half, apex not forming distinct sclerotized projection. Pygopods short, subconical, each with 16–20 erect spines.

###### Diagnosis.

*Eleodes pilosus* larvae can be separated from the other currently known *Eleodes* species by the pentagonal hypopharyngeal sclerome, lack of a caudal tooth on the pygidium, presence of 8–14 marginal spines on the pygidium, subquadrate prothoracic tergum, and having abdominal sternite I moderately clothed in long erect setae to posterior pigmented band.

### Key to the late instar larvae of 13 *Eleodes* species

**Table:** 

1	Lateral margins of abdominal sternites I–IX pigmented, with golden tomentose setae (Fig. 13A)	*Eleodes caudiferus* LeConte
1’	Lateral margins of abdominal sternites I–IX unpigmented, lacking tomentose setae (Fig. 13B)	2
2 (1’)	Apex of pygidium attenuated and sclerotized, forming a distinct projection (Fig. 14A)	3
2’	Apex of pygidium somewhat attenuated, lightly or unsclerotized, not forming distinct projection (Fig. 14B)	5
3 (2)	Anterolateral margins of epipharynx with micro-setation (Figs 8, 9A–B, D, 10A–D), lacking stout setae	*Eleodes armatus* LeConte
3’	Anterolateral margins of epipharynx with stout setae (Fig. 9C)	4
4 (3)	Mesotarsus with row of three ventromedial spines; ventral portion epicranial plates with row of six or more long setae along anterior margin near buccal cavity, confluent with setae on lateral portions of plates; prothoracic femur with ventromedial row of 13–14 spines	*Eleodes tenuipes* Casey
4’	Mesotarsus with two ventromedial spines, ventral portion epicranial plates with row of four to five long setae along anterior margin, not confluent with setae on lateral portions of plates; prothoracic femur with ventromedial row of 6–10 spines	*Eleodes hispilabris* (Say)
5 (2’)	Granulated band along lateral margins of protergum faint, concolorous with protergal disc (Fig. 7A)	6
5’	Granulated band along lateral margins of protergum distinct, darker than protergal disc (Fig. 7B–C)	8
6 (5)	Hypopharyngeal sclerome pentagonal, tricuspidate (Fig. 12B); ligula apex lacking microsetae, two long subapical setae present ventrally, eight or more subapical setae present dorsally (Fig. 11B); pigmented band present along posterior margin of abdominal sterna, integument usually tan	*Eleodes wheeleri* Aalbu, Smith, & Triplehorn
6’	Hypopharyngeal sclerome trapezoidal (Fig. 12A); ligula apex glabrous, four long subapical setae present, two ventrally and two dorsally (Fig. 11A); pigmented band absent along posterior margin of abdominal sterna, integument usually dark brown	7
7 (6)	Terga dark brown, nearly black, throughout; prothoracic tergum wider than long, 1.2× or more length of meso- or metaterga; posterior pigmented band on abdominal terga I–VIII darker than rest of segment; abdominal sternites nearly concolorous with tergites	*Eleodes carbonarius* (Say)
7’	Terga medium brown, lighter towards margins; prothoracic tergum subquadrate, 1.5× length of meso- or metaterga; posterior pigmented band on abdominal terga I–VIII concolorous with rest of segment; abdominal sternites lighter than tergites	*Eleodes anthracinus* (Blaisdell)
8 (5’)	Pigmented band around posterior margin of abdominal segments dark along anterior edge, fading to segment color posteriorly (Fig. 4A)	*Eleodes nigropilosus* (LeConte)
8’	Pigmented band around posterior margin of abdominal segments unicolorous, darker than rest of segment throughout	9
9 (8’)	Abdominal sternite I moderately clothed in long erect setae to posterior pigmented band; seven anterior sensory papillae present on epipharnyx in all specimens examined	*Eleodes pilosus* (Horn)
9’	Abdominal sternite I with sparsely setose on at most anterior half; six anterior sensory papillae present on epipharnyx in all specimens examined	10
10 (9’)	Antennal segment I longer than antennal segment II (Fig. 7A–B); gula length less than maximum width	*Eleodes extricatus* (Say)
10’	Antennal segment I subequal to antennal segment II (Fig. 7C); gula length subequal or greater than maximum width	11
11 (10’)	Pygidium with marginal row of 8–14 socketed spines; prothoracic tergum subquadrate, 1.5× length of meso- or metaterga	*Eleodes tribulus* Thomas
11’	Pygidium with marginal row of 18–20 socketed spines; prothoracic tergum wider than long, 1.2× or more length of meso- or metaterga	12
12 (11’)	Eighth and ninth abdominal tergites more darkly pigmented than preceding segments, protibia with ventromedial row of 3–4 spines	*Eleodes goryi* Solier
12’	Eighth and ninth abdominal tergites with same pigmentation as preceding segments; protibia with ventromedial row of 5–6 spines	*Eleodes subnitens* LeConte

### Notes on additional species

***Eleodes spinipes* (Solier).** One specimen of *Eleodes spinipes ventricosus* (TB08942) was reared to a late instar, 9th or 10th, in the lab. However, the specimen apparently died in its rearing container while molting and suffered some damage, thus obscuring many characters. What could be seen of the epipharnyx, ligula, and abdominal segment IX, including the presence of an apical tooth on the pygidium, place it with *Eleodes armatus*, *Eleodes hispilabris*, and *Eleodes tenuipes* in the subgenus *Eleodes*. The presence of spinose setae along the anterolateral margins of the epipharnyx placed it closest to *Eleodes hispilabris*, and *Eleodes tenuipes*.

### Previously described species

***Eleodes dentipes* Eschscholtz.** Little comparative data to separate the species can be drawn from Gissler (1878). Blaisdell (1909) provides a more detailed description, but likewise does not include many characters currently necessary to differentiate the species.

***Eleodes giganteus* Mannerheim.** Little comparative data to separate this species from the other currently described *Eleodes* larvae can be drawn from Gissler (1878).

***Eleodes pimelioides* (Mannerheim).** Little comparative data to separate this species from the other currently described *Eleodes* larvae can be drawn from Hyslop (1912).

***Eleodes suturalis* (Say).** The thorough description in Wade and St. George (1923) easily places this species within the subgenus *Eleodes* based on the epipharnyx and the apical tooth on the pygidium. It also appears to have spinose setae on the anterolateral margins of the epipharnyx, as in *Eleodes hispilabris*, *Eleodes spinipes*, and *Eleodes tenuipes*.

***Eleodes tricostatus* (Say).** McColloch (1918) describes the larvae as black in color after the first instar. The larvae of *Eleodes carbonarius* and *Eleodes anthracinus*, the only other *Melaneleodes* larvae known, are similarly dark. The picture provided by McColloch (plate 5, image B), also looks similar to *Eleodes carbonarius* in gestalt.

***Eleodes vandykei* Blaisdell.** Little comparative data to separate this species from the other currently described *Eleodes* larvae can be drawn from Hyslop (1912).

## Discussion

As adult morphology in many *Eleodes* species may be heavily influenced by participation in mimicry rings with co-occurring species (Doyen and Somerby 1974), the addition of characters from larval morphology may help produce a more accurate phylogeny based on morphological data than one using adult morphology alone. The presented phylogeny (Fig. 1) demonstrates the utility of larval morphology in resolving at least some relationships within the genus *Eleodes*. The subgenus *Melaneleodes* was well supported based on several synapomorphies present in the two included species. The subgenus *Eleodes* showed two synapomorphies supporting a relationship for three of the included species, but did not recover a clade containing all of the current or presumed species from the nominate subgenus. Both *Eleodes tribulus* and *Eleodes caudiferus* are somewhat unusual members of the subgenus based on adult morphology as well; hence further research is needed to accurately place them within the subgeneric classification. The inclusion of more taxa should increase phylogenetic accuracy and help illuminate the currently unresolved relationships between the *Eleodes* subgenera (Heath et al. 2008). It is likely that extensive modifications to the matrix and key will be needed as more larvae become known. It is also possible that some species, particularly closely related ones, cannot be separated based on larval characters alone.

By producing matrix-based descriptions within mx, we are creating a growing repository of digital morphological and specimen data, already available through the tenebrionidbase.org portal, including an online multi-entry key (http://tenebrionidbase.org/public/clave) to the currently known *Eleodes* larvae. Characters and states from the matrix and key will also be linked to the developing Coleoptera Anatomy Ontology project (ColAO).

## Supplementary Material

XML Treatment for Eleodes

XML Treatment for Eleodes (Blapylis) nigropilosus

XML Treatment for Eleodes (Caverneleodes) wheeleri

XML Treatment for Eleodes (Eleodes) armatus

XML Treatment for Eleodes (Eleodes) caudiferus

XML Treatment for Eleodes (Eleodes) hispilabris

XML Treatment for Eleodes (Eleodes) tenuipes

XML Treatment for Eleodes (Eleodes) tribulus

XML Treatment for Eleodes (Litheleodes) extricatus

XML Treatment for Eleodes (Melaneleodes) anthracinus

XML Treatment for Eleodes (Melaneleodes) carbonarius knausii

XML Treatment for Eleodes (Promus) goryi

XML Treatment for Eleodes (Promus) subnitens

XML Treatment for Eleodes (Tricheleodes) pilosus

## Appendix 1

1. Head – orientation: (0) prognathous or weakly declined; (1) hypognathous
2. Head – shape: (0) rounded; (1) weakly dorsoventrally flattened; (2) strongly dorsoventrally flattened
3. Head – width: (0) narrower than prothorax; (1) nearly equal to prothorax; (2) wider than prothorax
4. Head – sides: (0) rounded; (1) angular
5. Head – constriction: (0) strongly constricted before occipital foramen; (1) weakly constricted before occipital foramen
6. Head – color: (0) medium brown; (1) brown–grey; (2) ferruginous; (3) dark brown; (4) light tan; (5) dark tan
7. Head – color vs body color: (0) same or nearly the same as body segments; (1) more heavily pigmented than body segments
8. Head – punctation: (0) impunctate; (1) minute; (2) moderate
9. Head – punctation density: (0) impunctate; (1) sparse, separated by more than 4 puncture diameters; (2) moderate, separated by 2–4 puncture diameters; (3) dense, separated by 1–2 puncture diameters; (4) nearly confluent, separated by less than a puncture diameter; (5) confluent, at least some punctures merged
10. Epicranial suture – stem length: (0) approximately one–third head capsule length; (1) approximately one–fourth head capsule length
11. Epicranial suture – frontal arms: (0) U–shaped, not obscured by sculpturing; (1) U–shaped, partially obscured by sculpturing; (2) V–shaped, not obscured by sculpturing; (3) V–shaped, obscured by sculpturing
12. Frons – sculpturing: (0) smooth; (1) faintly rugose; (2) rugose
13. Epicranial plates – dorsal sculpturing: (0) smooth; (1) faintly rugose; (2) rugose
14. Frons – non–primary setae: (0) absent; (1) present
15. Lateral portions of epicranial plates: (0) sparsely setose; (1) moderately setose; (2) densely setose
16. Ventral portions of epicranial plates – setation 1: (0) with row of four to five long setae along anterior margin near buccal cavity, not confluent with setae on lateral portions of plates; (1) with row of six or more long setae along anterior margin near buccal cavity confluent with setae on lateral portions of plates; (2) with two long setae along anterior margin near buccal cavity, not confluent with setae on lateral portions of plates
17. Ventral portions of epicranial plates – setation 2: (0) patch of short setae medially forming a triangular pattern with its base near the anterior margin
18. Stemmata: (0) two present on each epicranial plate, pigmented spots often faded
19. Clypeus – shape: (0) trapezoidal
20. Clypeus – inflation: (0) not swollen; (1) swollen
21. Clypeus – punctation density: (0) impunctate; (1) sparse, separated by more than 4 puncture diameters; (2) moderate, separated by 2–4 puncture diameters; (3) dense, separated by 1–2 puncture diameters; (4) nearly confluent, separated by less than a puncture diameter; (5) confluent, at least some punctures merged
22. Clypeus – pigmentation: (0) unicolorous; (1) darker medially in basal half; (2) basal half darker; (3) apical half darker
23. Labrum – inflation: (0) not swollen; (1) swollen
24. Labrum – pigmentation: (0) unicolorous; (1) basal half more darkly pigmented
25. Labrum – sides: (0) rounded; (1) straight
26. Labrum – medial setal row: (0) absent; (1) six to seven erect setae; (2) seven to eight erect setae; (4) ten to fourteen erect setae; (5) four erect setae; (6) two erect setae
27. Labrum – subapical setal row: (0) absent; (1) six to seven erect setae; (2) seven to eight erect setae; (4) ten to fourteen erect setae
28. Labrum – anterior margin: (0) straight to weakly emarginate; (1) medially emarginate
29. Epipharynx – anterior setal row: (0) absent; (1) with six stout spiniform setae; (2) with eight or more stout spiniform setae
30. Epipharynx – anterolateral margins: (0) with stout spinose setae; (1) with micro–setation; (2) lacking setation
31. Epipharnyx – anterior sensory papillae (spinule) number: (0) six; (1) seven; (2) eight; (3) four; (4) five
32. Epipharnyx – anterior sensory papillae (spinule) arrangement: (0) two irregular diagonal rows; (1) two irregular longitudinal rows; (2) two irregular rows, each with two posterior papillae and one near the anterior margin; (3) irregular cluster
33. Epipharnyx – subanterior sensory papillae: (0) transverse row of four small sensory papillae subtended by two spinose setae
34. Epipharnyx – posterior sensory papillae number: (0) six; (1) seven; (2) eight
35. Epipharnyx – posterior sensory papillae arrangement: (0) two irregular rows; (1) irregular cluster
36. Tormae: (0) strongly asymmetric; (1) weakly asymmetric
37. Tormae 2: (0) symmetrical; (1) asymmetrical, left torma smaller; (2) asymmetrical, left torma larger; (3) asymmetrical, left torma smaller, with or without small spine near emergent edge
38. Ligula – setae: (0) apex glabrous, four long subapical setae present, two ventrally and two dorsally; (1) apex densely microsetose, two long subapical setae present ventrally; (2) apex lacking microsetae, two long subapical setae present ventrally, eight or more subapical setae present dorsally; (3) apex with fringe of 6–10 long setae, medially with longitidinal row of short stout setae; (4) apex with median longitudinal row of microsetae dorsally, two long subapical setae present ventrally
39. Hypopharyngeal sclerome: (0) trapezoidal; (1) pentagonal, tricuspidate; Shape of hypopharyngeal sclerome
40. Gula: (0) distinct, hexagonal, widest near middle; (1) distinct, weakly trapezoidal, nearly rectangular; (2) trapezoidal, widest at base
41. Gula – fusion: (0) sutures visible throughout; (1) sutures fused, not visible in basal half
42. Gula – length: (0) less than maximum width; (1) subequal or greater than maximum width
43. Antenna: (0) three segmented, cylindrical, first segment longer than second; (1) three segmented, cylindrical, first segment subequal to second; (2) three segmented, cylindrical, first segment shorter than second
44. Antenna – segment 2 sensorium: (0) forming a single incomplete ring around the base of segment 3; (1) consisting of many small rounded sensoria around base of segment 3
45. Thoracic tergites – color: (0) grey–brown; (1) medium brown; (2) ferruginous; (3) dark brown; (4) light tan
46. Prothoracic sternite – color anterior to legs: (0) grey–brown; (1) medium brown; (2) ferruginous; (3) dark brown; (4) light brown
47. Thoracic sternite color – posterior to prolegs: (0) tan; (1) light brown; (2) medium brown
48. Prothoracic tergum – anterior transverse striated band: (0) absent; (1) present along anterior fourth, lighter than tergal disc; (2) present along anterior fourth, darker than tergal disc
49. Thoracic tergites – posterior transverse band: (0) absent; (1) present along posterior 5th of all thoracic tergites, striated; (2) present along posterior 6th of all thoracic tergites; striated (3) present along posterior 4th of all thoracic tergites, striated; (4) present, not striated
50. Thoracic tergites – posterior striated band color: (0) absent; (1) forming a gradient from darker brown anteriorly to lighter brown along posterior border; (2) unicolorous, brown, darker midtergite
51. Mesothoracic tergite – sclerotized transverse line: (0) absent; (1) present on anterior fifth, heavily sclerotized; (2) present on anterior fifth, faint
52. Metathoracic tergite – sclerotized transverse line: (0) absent; (1) present on anterior fifth, heavily sclerotized; (2) present on anterior fifth, faint
53. Thoracic tergites – setae: (0) absent; (1) eight evenly arranged setae present on dorsal surface of each thoracic tergite, lateral margins more densely setose; (2) more than eight dorsal setae present, pattern variable; (3) dense transverse band of short setae near anterior margins of meso– and metatergites
54. Prothoracic tergum – shape: (0) subquadrate, 1.5× or more length of meso– or metaterga; (1) wider than long, 1.2× or more length of meso– or metaterga
55. Prothoracic tergum – lateral margins: (0) granulated band faint, concolorous with protergal disc; (1) granulated band distinct, darker than protergal disc; (2) granulated band absent
56. Meso– and metaterga – shape: (0) wider than long
57. Meso– and metaterga – lateral margin: (0) lacking pigmented bands; (1) pigmented bands present
58. Mesothoracic spiracle: (0) simple, ovate, approximately 1.5× size of abdominal spiracles
59. Prothoracic legs: (0) slightly longer and much thicker than meso– and metathoracic legs; (1) slightly longer and slightly thicker than meso– and metathoracic legs
60. Prothoracic legs – tarsungulus: (0) strongly sclerotized and sickle–shaped; (1) strongly sclerotized, attenuated and slightly hooked
61. Prothoracic legs – trochanter: (0) with row of two stout spines and two longer setae ventro–medially; (1) with row of two stout spines ventro–medially; (2) one stout ventro–medial spine present
62. Prothoracic legs – femur: (0) with ventro–medial row of two spines and three to five longer setae; (1) with ventro–medial row of three to four spines and four to five longer setae; (2) with ventro–medial row of five to six spines; (3) with ventro–medial row of six to ten spines; (4) with ventro–medial row of 13 to 14 spines; (5) with ventro–medial row of three spines; (6) with ventro–medial row of four spines
63. Prothoracic legs – tibia: (0) with ventro–medial row of three to four spines; (1) with ventro–medial row of five to six spines; (2) with ventro–medial row of eight to eleven spinose setae; (3) with eight or more spines ventro–medially, not forming a regular row
64. Prothoracic legs – femur dorsal surface (at rest): (0) with faintly indicated basal sclerotized band; (1) lacking basal sclerotized band
65. Prothoracic legs – tibia dorsal surface (at rest): (0) slightly more sclerotized than ventral surface
66. Mesotibia – posterior surface: (0) row of three spines; (1) row of two spines; (2) row of four to five spines; (3) row of five to seven spinose setae; (4) four spines in 2×2 pattern
67. Abdominal tergites 1–7 – color: (0) light tan; (1) grey–brown; (2) medium brown; (3) ferruginous; (4) dark brown
68. Abdominal tergites 1–7 – color gradation: (0) unicolorous; (1) lightening towards lateral margins; (2) darkening towards lateral margins
69. Abdominal sternites – color: (0) light tan; (1) dark tan; (2) ferruginous
70. Abdominal tergites 1–8 – transverse striated bands: (0) absent; (1) barely visible on posterior 5th of segments; (2) distinct on posterior 5th of segments
71. Abdominal sternites 1–8 – transverse striated bands: (0) absent; (1) barely visible on posterior 5th of segments; (2) distinctly visible, forming near contiguous band with tergal band
72. Abdominal sternite I – setae: (0) absent; (1) sparsely clothed in long erect setae from anterior margin to near midline; (2) moderately clothed in long erect setae to posterior pigmented band; (3) moderately clothed in long erect setae from anterior margin to near midline; (4) tomentose in anterior third, denser along near lateral margins; (5) sparsely clothed in long erect setae along anterior margin
73. Abdominal segments 2–8 – setae: (0) absent; (1) each segment with two sparse transverse bands of long erect setae; (2) each segment with two sparse transverse bands of long erect setae, posterior margin of segment 8 denser setal band; (3) otherwise
74. Abdominal tergites 1–8 – posterior margin color gradation: (0) dark along anterior edge, fading to segment color posteriorly; (1) unicolorous, darker than rest of segment throughout
75. Abdominal tergite 8 – pigmentation: (0) more darkly pigmented than preceding segments; (1) same pigmentation as preceding segments
76. Abdominal tergite 9 – pigmentation: (0) more darkly pigmented than preceding segments; (1) same pigmentation as preceding segments; (2) lighter than preceding segments
77. Abdominal laterotergites: (0) concolorous with tergites, lacking distinct pigmented margins; (1) lateral margins distinctly pigmented
78. Abdominal segment IX, pygidium – dorsal aspect: (0) triangular in dorsal view, gradually reflexed to apex, apex not forming a distinct tooth; (1) triangular in dorsal view, gradually reflexed to apex, apex attenuated and sclerotized, forming a distinct tooth; (2) triangular in dorsal view, gradually reflexed to apex, apex attenuated and sclerotized, rarely forming a small tooth in some specimens; (3) triangular in dorsal view, gradually reflexed to apex, apex with distinct urigomphi
79. Abdominal segment IX, pygidium – setae: (0) absent; (1) sparsely clothed in short and mid length erect setae; (2) moderately clothed in short and mid length erect setae; (3) few primary setae
80. Abdominal segment IX, pygidium – dorsal sculpturing: (0) dorsally more sclerotized in apical two–thirds with faint maculations; (1) sclerotization uniform throughout, lacking maculations
81. Abdominal segment IX, pygidium – marginal row of socketed spines: (0) 14–18 spines; (1) 18–20 spines; (2) 17–23 spines; (3) 22–24 spines; (4) 8–14 spines; (5) 27 spines; (6) 28–38 spines; (7) four spines
82. Abdominal segment IX, pygidium – marginal row of socketed spines 2: (0) forming a single row around posterior two–thirds to one half of segment; (1) forming two or three irregular rows around posterior two–thirds to one half of segment, narrowing to single row around apex; (2) four distinct, regular spines
83. Abdominal sternites I–VIII, longitudinal tomentose bands along lateral margins: (0) present; (1) absent
84. Abdominal segment X – pygopods: (0) short, subconical; (1) longer, tip inverted
85. Abdominal segment X – pygopod setation: (0) each with 9–12 erect setae; (1) each with 11–15 erect setae; (2) each with 17–24 erect setae; (3) each with 10 or more erect setae onmore heavily sclerotized posterior face
86. Urogomphi: (0) absent; (1) present, connected at base or complex; (2) present, paired

## Appendix 2

Descriptive Character Codings

**Table:** Characters with “{ }” indicate polymorphic codings

Eleodes_nigropilosus	01100501202110100000210101201100021111100110411211221010100015000100022120111021001000
Eleodes_tribulus	01110{04}112022201100012111011011{04}{03}0{12}1021100110411212221010100010000000022321111021401010
Eleodes_wheeleri	0110040130211001000121110120113102111211001044{01}212221100000016100200022511110211001010
Eleodes_armatus	01100211312110110001211102201102021012100000{24}212121111101000132003{03}0{02}1{12}311111111301010
Eleodes_caudiferus	010005012{01}22202100012{01}11044021000211011000102212120030101000121000{03}0022431111221610020
Eleodes_hispilabris	010002113122201000013111014020020{12}10221000004{24}12121111101000{12}3210200022121111111201000
Eleodes_tenuipes	0{01}1002113122201100013{12}11014020020210121000004212121211101000242103000221111111215010?0
Eleodes_extricatus	0110040130211011000{01}3011011011000211{01}1100000421212111010100010000200022121111011201010
Eleodes_anthracinus	01000{01}01200110100000210102201100020010000000{01}{01}01111110000000000000{12}1{01}1{01}120110010{014.0}01010
Eleodes_carbonarius_knausi	01000{23}01200110100000210101101100021120000000{23}{23}11221111000000111002{34}0{01}20121120010101000
Eleodes_goryi	01100{23}112001101100012111011011000210211001104{12}22{23}2111110100010000000022321001020101010
Eleodes_subnitens	01100211202110110001231101201100021021100110421232121110100010100200022221111021101020
Eleodes_pilosus	01000{45}0131122121000120110{12}{12}01111011031100000412232222110100012100100022211111021201020
Tenebrio_molitor	01100201302110020001311006202000021014120120222232111010101115000400022511001331721132
Zophobas_morio	01100{23}114122201000013211051012010211131210211122420010001011113102301215000010307211?0

## Appendix 3

**Cladistic Morphological Character Matrix**

1. Head – width: (0) nearly equal to prothorax; (1) narrower than prothorax
2. Head – color vs body color: (0) more heavily pigmented than body segments; (1) same or nearly the same as body segments
3. Head – punctation density: (0) impunctate-nearly confluent, separated by less than a puncture diameter; (1) sparse, separated by more than 4 puncture diameters; (2) moderate, separated by 2-4 puncture diameters; (3) dense, separated by 1-2 puncture diameters
4. Epicranial suture – stem length: (0) approximately one third head capsule length; (1) approximately one fourth head capsule length
5. Epicranial suture – frontal arms: (0) sinuate; (1) U shaped
6. Frons – sculpturing: (0) distinctly rugose; (1) faintly rugose
7. Epicranial plates – dorsal sculpturing: (0) distinctly rugose; (1) faintly rugose
8. Lateral portions of epicranial plates: (0) sparse to moderately setose; (1) densely setose
9. Ventral portions of epicranial plates – setation: (0) with row of four to five long setae along anterior margin near buccal cavity, not confluent with setae on lateral portions of plates; (1) with row of six or more long setae along anterior margin near buccal cavity, confluent with setae on lateral portions of plates; (2) with two long setae along anterior margin near buccal cavity, not confluent with setae on lateral portions of plates
10. Clypeus – shape: (0) swollen; (1) not swollen
11. Clypeus – punctation density: (0) dense, separated by 1-2 puncture diameters; (1) moderate, separated by 2-4 puncture diameters
12. Labrum – inflation: (0) swollen; (1) not swollen
13. Labrum – subapical setal row: (0) six to seven erect setae; (1) seven to eight erect setae; (2) ten to fourteen erect setae
14. Epipharynx – anterior setal row: (0) with six stout spiniform setae; (1) with eight or more stout spiniform setae
15. Epipharynx – anterolateral margins: (0) lacking setation; (1) with stout spinose setae; (2) with micro-setation
16. Epipharnyx – anterior spinule arrangement: (0) two semi uniform rows or irregular cluster; (1) two rows, each with two posterior papillae and one near the anterior margin
17. Tormae: (0) weakly asymmetric, more irregularly shaped and somewhat acute; (1) strongly asymmetric, broadly triangular and acute
18. Ligula – setae: (0) apex with fringe of 6-10 long setae medially with longitudinal row of short stout setae; (1) apex with median longitudinal row of microsetae dorsally two long subapical setae present ventrally; (2) apex densely microsetose two long subapical setae present ventrally; (3) apex lacking microsetae, two long subapical setae present ventrally, eight or more subapical setae present dorsally; (4) apex glabrous, four long subapical setae present two ventrally and two dorsally
19. Hypopharyngeal sclerome: (0) pentagonal, tricuspidate; (1) trapezoidal
20. Gula: (0) trapezoidal, widest at base; (1) distinct, hexagonal to nearly rectangular, widest near middle
21. Gula – length: (0) equal to or less than maximum width; (1) greater than maximum width
22. Antennae: (0) three segmented, cylindrical, first segment shorter than second; (1) three segmented, cylindrical, first segment longer than second; (2) three segmented, cylindrical, first segment subequal to second
23. Prothoracic tergum – anterior transverse striated band: (0) present along anterior fourth, darker than tergal disc; (1) present along anterior fourth, lighter than tergal disc
24. Mesothoracic tergite – sclerotized transverse line: (0) absent; (1) present on anterior fifth, heavily sclerotized; (2) present on anterior fifth, faintly indicated
25. Metathoracic tergite – sclerotized transverse line: (0) absent; (1) present on anterior fifth, heavily sclerotized; (2) present on anterior fifth, faintly indicated
26. Thoracic tergites – setae: (0) eight evenly arranged setae present on dorsal surface of each thoracic tergite, lateral margins more densely setose; (1) more than eight dorsal setae present, pattern variable
27. Prothoracic tergum – shape: (0) subquadrate, 1.5× length of meso- or metaterga; (1) wider than long, 1.2× or more length of meso- or metaterga
28. Prothoracic tergum – lateral margins: (0) granulated band faint, concolourous with protergal disc; (1) granulated band distinct, darker than protergal disc; (2) granulated band absent
29. Meso- and metaterga – lateral margin: (0) pigmented bands present; (1) lacking pigmented bands
30. Prothoracic legs: (0) slightly longer and slightly thicker than meso- and metathoracic legs; (1) slightly longer and much thicker than meso- and metathoracic legs
31. Prothoracic legs – tarsungulus: (0) strongly sclerotized, attenuated and slightly hooked; (1) strongly sclerotized and sickle shaped
32. Prothoracic legs – tibia: (0) with ventro–medial row of three to four spines; (1) with ventro-medial row of five to six spines; (2) with ventro–medial row of eight to eleven spinose setae; (3) with eight or more spines ventro–medially not forming a regular row
33. Prothoracic legs – femur dorsal surface at rest: (0) with faintly indicated basal sclerotized band; (1) lacking basal sclerotized band
34. Abdominal sternites – color: (0) light tan; (1) dark tan; (2) ferruginous
35. Abdominal sternites I-VIII – transverse striated bands: (0) barely visible on posterior 5th of segments; (1) distinctly visible, forming near contiguous band with tergal band
36. Abdominal sternite I – setae: (0) sparsely clothed in long erect setae along anterior margin; (1) sparsely clothed in long erect setae from anterior margin to near midline; (2) moderately clothed in long erect setae to posterior pigmented band; (3) moderately clothed in long erect setae from anterior margin to near midline; (4) tomentose in anterior third, denser along near lateral margins
37. Abdominal segments II-VIII – setae: (0) absent; (1) each segment with two sparse transverse bands of long erect setae; (2) each segment with two sparse transverse bands of long erect setae, posterior margin of segment 8 denser setal band; (3) otherwise
38. Abdominal tergites I-VIII – posterior margin color gradation: (0) dark along anterior edge, fading to segment color posteriorly; (1) unicolorous darker than rest of segment throughout
39. Abdominal tergite VIII – pigmentation: (0) more darkly pigmented than preceding segments; (1) same pigmentation as preceding segments
40. Abdominal tergite IX – pigmentation: (0) more darkly pigmented than preceding segments; (1) same pigmentation as preceding segments; (2) lighter than preceding segments
41. Abdominal laterotergites: (0) lateral margins distinctly pigmented; (1) concolorous with tergites, lacking distinct pigmented margins
42. Abdominal segment IX (pygidium) – shape: (0) triangular in dorsal view, gradually reflexed to apex, apex not forming a distinct tooth; (1) triangular in dorsal view, gradually reflexed to apex, apex attenuated and sclerotized forming a distinct tooth; (2) triangular in dorsal view, gradually reflexed to apex, apex attenuated and sclerotized rarely forming a small tooth in some specimens; (3) triangular in dorsal view, gradually reflexed to apex, apex with distinct urigomphi
43. Abdominal segment IX (pygidium) – setae: (0) few primary setae; (1) sparsely clothed in short and mid-length erect setae; (2) moderately clothed in short and mid-length erect setae
44. Abdominal segment IX (pygidium) – dorsal sculpturing: (0) dorsally more sclerotized in apical two thirds, with faint maculations; (1) sclerotization the same throughout, lacking maculations
45. Abdominal segment IX (pygidium) – marginal row of socketed spines: (0) four; (1) eight or more
46. Abdominal segment IX (pygidium) – marginal row of socketed spines 2: (0) four distinct regular spines; (1) forming a single row around posterior two thirds to one half of segment; (2) forming two or three irregular rows around posterior two thirds to one half of segment, narrowing to single row around apex
47. Abdominal segment X – pygopods: (0) longer, tip inverted; (1) short, subconical
48. Abdominal segment X – pygopod setation: (0) each 16 or less setae; (1) each with 17 or more setae

## Appendix 4

**Table:** Cladistic Character Codings

Eleodes_nigropilosus	012001100111102002011202200101100011201100211110
Eleodes_tribulus	002000001010002012011202200101100013211100211110
Eleodes_wheeleri	013001101010102003010202201011110010111112111110
Eleodes_armatus	003101101010102113010101101101120213111101111110
Eleodes_caudiferus	112100011010212002010200010101110014311102211210
Eleodes_hispilabris	103100000000211113010101101101121011211101111111
Eleodes_tenuipes	00310000100021111301010120110112101111110121111?
Eleodes_extricatus	013001101000002002010101100101100011211100111110
Eleodes_anthracinus	112011100111102014110111100011100101201110101110
Eleodes_carbonarius_knausi	112011100111002004110111101011110101211210101110
Eleodes_goryi	002011101010002012011201101101100013210000201110
Eleodes_subnitens	002001101010102012011201201101110012211100211111
Eleodes_pilosus	113110011010[01]02012010102211101110012111100211111
Tenebrio_molitor	013001102000111011001001100100000010110003010000
Zophobas_morio	000100000000000000000000000000031100000000000000
